# Microbiome engineering optimized by Antarctic microbiota to support a plant host under water deficit

**DOI:** 10.3389/fpls.2023.1241612

**Published:** 2023-09-15

**Authors:** Rodrigo Rodríguez, Patricio J. Barra, Giovanni Larama, Víctor J. Carrion, María de la Luz Mora, Lauren Hale, Paola Durán

**Affiliations:** ^1^ Programa de Doctorado en Ciencias de Recursos Naturales, Universidad de La Frontera, Temuco, Chile; ^2^ Biocontrol Research Laboratory, Universidad de La Frontera, Temuco, Chile; ^3^ Agroscientific SpA, Temuco, Chile; ^4^ Center of Plant, Soil Interaction and Natural Resources Biotechnology, Scientific and Technological Bioresource Nucleus, Universidad de La Frontera, Temuco, Chile; ^5^ Institute of Biology, Leiden University, Leiden, Netherlands; ^6^ USDA, Agricultural Research Service, San Joaquin Valley Agricultural Sciences Center, Parlier, CA, United States; ^7^ Facultad de Ciencias Agropecuarias y Medioambiente, Departamento de Producción Agropecuaria, Universidad de La Frontera, Temuco, Chile

**Keywords:** Antarctic microbiome, sustainable agriculture, climate change, microbiome transplant, water deficit stress, Host Mediated Microbiota Selection (HMMS), extreme environment

## Abstract

Climate change challenges modern agriculture to develop alternative and eco-friendly solutions to alleviate abiotic and/or biotic stresses. The use of soil microbiomes from extreme environments opens new avenues to discover novel microorganisms and microbial functions to protect plants. In this study we confirm the ability of a bioinoculant, generated by natural engineering, to promote host development under water stress. Microbiome engineering was mediated through three factors i) Antarctic soil donation, ii) water deficit and iii) multigenerational tomato host selection. We revealed that tomato plants growing in soils supplemented with Antarctic microbiota were tolerant to water deficit stress after 10 generations. A clear increase in tomato seedling tolerance against water deficit stress was observed in all soils over generations of Host Mediated Microbiome Engineering, being Fildes mixture the most representatives, which was evidenced by an increased survival time, plant stress index, biomass accumulation, and decreased leaf proline content. Microbial community analysis using 16s rRNA gene amplicon sequencing data suggested a microbiome restructuring that could be associated with increased tolerance of water deficit. Additionally, the results showed a significant increase in the relative abundance of *Candidatus* Nitrosocosmicus and *Bacillus* spp. which could be key taxa associated with the observed tolerance improvement. We proposed that *in situ* microbiota engineering through the evolution of three factors (long-standing extreme climate adaption and host and stress selection) could represent a promising strategy for novel generation of microbial inoculants.

## 1 Introduction

It is estimated that by 2050 the world population will exceed 9 billion people and that crop productivity should increase by 70% to meet food demands (FAO et al., 2021). However, both the number and severity of drought events are predicted to increase in near future, especially from 2019–2034, impacting crop productivity (Spinoni et al., 2020; Waseem et al., 2021). This is critical for plants with high water demand, such as tomato, which require between 400 and 600 mm of irrigation water for seasonal production (Ronga et al., 2019a; Ronga et al., 2019b). Therefore, it is imperative to develop sustainable strategies to optimize plant productivity and food quality under water scarcity.

Today, it is widely accepted that microorganisms have important roles in determining host fitness during periods of water deficit stress (Naylor et al., 2017; Xu and Coleman-Derr, 2019). Many bacteria from the endosphere and rhizosphere microbiome have been described and highlighted as providing a benefit to their host by several well-documented mechanisms and others that have not yet been fully clarified. A main bacterial mechanism is the production of the enzyme 1-aminocyclopropane-1-carboxylate (ACC) deaminase (ACCD), which catalyzes the hydrolysis of the immediate precursor of ethylene, the ACC, to ammonia and α-ketobutyrate (Tiwari et al., 2018). Many plants growth-promoting bacteria (PGPB), can also produce phytohormones such as indole acetic acid (IAA), which regulates various development processes in the plant, such as root initiation, stem elongation, apical dominance, root size and distribution, resulting in greater water and nutrient absorption from the soil (Orozco-Mosqueda et al., 2023). Traditionally, PGPB have been isolated, characterized, identified, bioaugmented and re-inoculated into soil or plant seeds, with the finality of optimizing plant fitness under a stressing condition, such as nutrient limitation or water stress (Barra et al., 2017; Barra et al., 2019; Rahnama et al., 2023). However, despite the promising findings of bacterial bioinoculants in laboratory and growth chamber conditions, their performance at the field level has frequently been suboptimal, resulting in limited commercial adoption on a large scale (O’Callaghan et al., 2022). Several hypotheses have been formulated to explain these dissimilarities and inconsistencies among controlled and field conditions, such as low inoculum survival due to competition with native microorganisms, starvation, limited distribution of the inoculant in the soil profile, and variations in soil properties, among other (Kaminsky et al., 2019). Furthermore, the utilization of inoculums based solely on individual bacteria may be constrained due to the intricate synergistic interactions and complex interconnected networks that exist among various components of the microbiota (Backer et al., 2018; Qiu et al., 2019). Auspiciously, some studies have showed that inoculation with bacterial consortia is a more effective approach than inoculation with a single strain, since bacteria seem to function synergistically and can along compete for certain ecological niches (Berendsen et al., 2018a; Woo and Pepe, 2018). For these reasons, the use *in situ* of all (or most) microbiota, their synergy and the complex networks, avoiding previous cultivation, could represent a promising alternative for breaking down the barriers of pre-existing microbial niches and, thus, more efficiently incorporate bioinoculants with beneficial properties for crops.

Plant-microbe relationships are often shaped by alterations in radical exudation of primary and secondary plant metabolites, such as organic acids, tryptophan and strigolactone, which can be substrates for microbial growth, elicit chemotactic responses, facilitate root colonization, or inhibit the growth of some specific microbial taxa (Rudrappa et al., 2008; Hassan and Mathesius, 2012; Liu et al., 2017; Massalha et al., 2017; Menezes-Blackburn et al., 2021). The process by which the plant modulates root exudation of metabolites depends on various intrinsic plant factors as well as external factors such as nutrient availability, management practices, abiotic stresses, soil conditions and the surrounding organisms (Compant et al., 2019; Rolfe et al., 2019; Menezes-Blackburn et al., 2021). In turn, plant-associated microorganisms can induce specific systemic changes in root exudation, and therefore also modulate the plant-associated microbiome structure (Korenblum et al., 2020).

In recent years, Host-Mediated Microbiome Engineering (HMME) has emerged as a novel and eco-friendly *in situ* microbiome engineering strategy based on the plant’s intrinsic ability to recruit and maintain a beneficial microbiome (Lakshmanan et al., 2012; Jochum et al., 2019; Mueller et al., 2019). By employing a host multigenerational approach and visualizing changes in the host phenotype, this strategy enables the indirect selection of a specific microbiome, wherein the plant recruits a diverse array of microorganisms. These microorganisms, encompassing beneficial, neutral, or potentially negative types, collectively contribute to the overall well-being and favorability of the plant (Swenson et al., 2000; Raaijmakers and Mazzola, 2016; Mathur and Roy, 2021). Although HMME has only recently been encompassed as a subject of study, this natural plant defense mechanism was evidenced in early reports of ‘suppressive soils’(Cook and Rovira, 1976; Jayaraman et al., 2021). Natural suppression of soil- borne pathogens were induced by crop monoculture (Wildermuth, 1982; Sturz et al., 1999; Cook, 2003; Sagova-Mareckova et al., 2015; Sahu et al., 2018), wherein plants ‘recruited’ microorganisms involved in disease protection after a pathogen outbreak, a phenomena called ‘*The Cry for Help*’ (Bakker et al., 2018; Berendsen et al., 2018b; Li et al., 2019; Rolfe et al., 2019).

We recently reported that HMME over multiple generations can be achieved by managing three co-occurring factors; i) a favorable microbiota as a donor of microorganisms, ii) a stressor factor to induce plant response and shape microbial community assemblages, iii) a host model as modulator (Durán et al., 2021). Extreme environments are an interesting habitat likely to contain microbiota with the potential to enhance plant fitness (Jeon et al., 2009; Mishra et al., 2011; Gonc et al., 2015; Peixoto et al., 2016) due to strong selective pressure. These environments may lead to the evolution of novel mechanisms for stress tolerance (Convey and Smith, 2006; Kasana and Gulati, 2011; Liu et al., 2019). In this way, Antarctica is a unique ‘laboratory’ to study life in extreme conditions, where the combination of an extensive glacial layer, intense katabatic winds, and extremely low precipitation rates makes them the oldest, coldest, and driest deserts on Earth (Rycroft, 1987). So far, several studies show that the Antarctic microorganisms could confer plants protection against different abiotic stresses (Gallardo-Cerda et al., 2018; Yarzábal et al., 2018; Garcia et al., 2021; Styczynski et al., 2022).

In this study, we investigated the effect of Antarctic microbiota donation and plant microbiome selection by HMME on the improvement of water deficit stress tolerance in tomato plants. We focused our study on addressing three major questions: (1) Is it possible to improve the tolerance of tomato plants to water deficit through Antarctic microbiota transference using HMME? (2) Can extreme microbiota survive in agricultural soil? (3) If so, which taxonomic groups co-evolve with the host-plant during the multigenerational water deficit selection? By answering these questions, we hope to contribute new approaches to enhance sustainable agriculture through the development of a new generation of *in situ* bioinoculants.

## 2 Materials and methods

### 2.1 Host-mediated microbiome engineering on tomato plants exposed to water deficit

**Figure 1:**
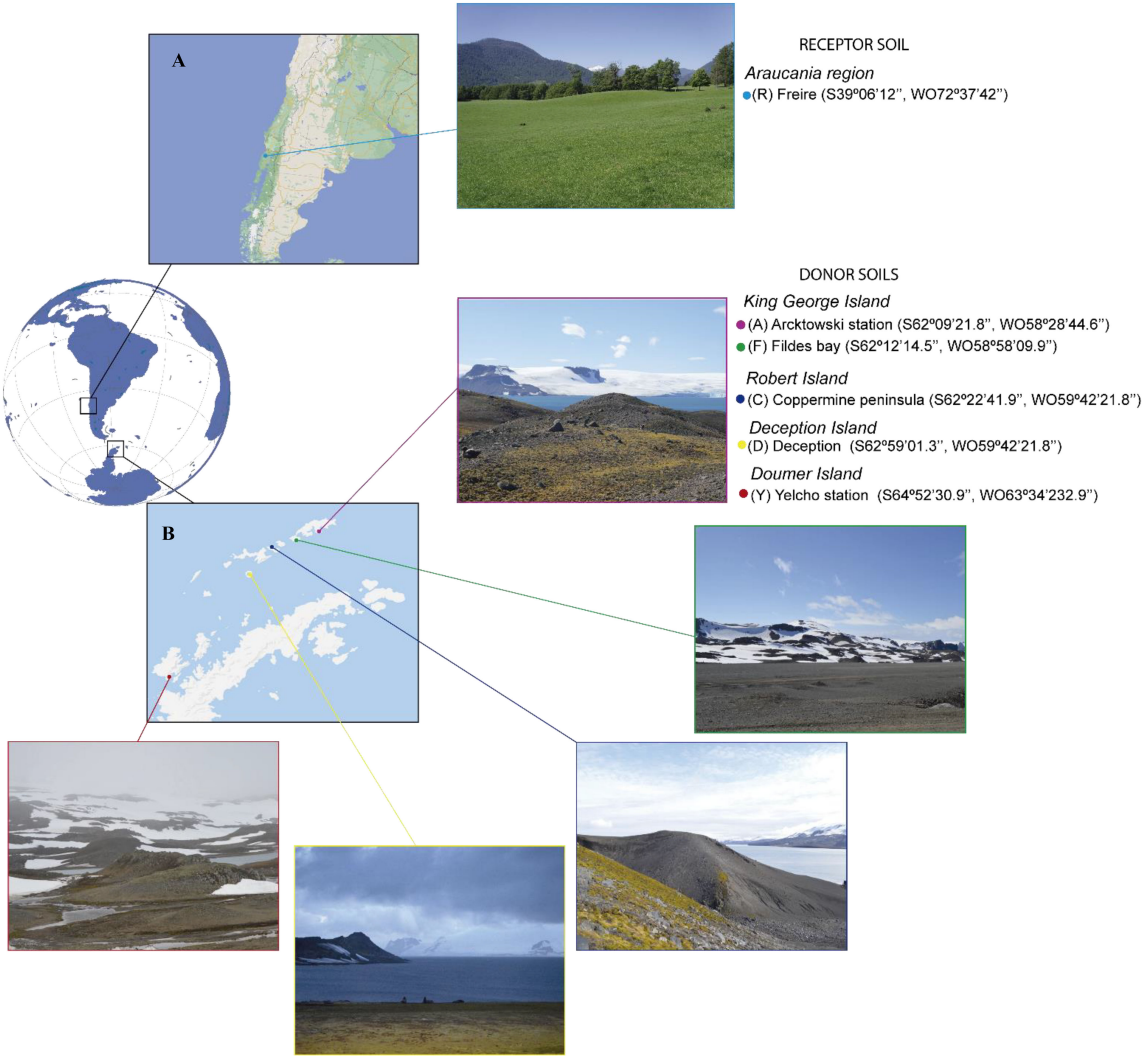
Soil Sampling. **(A)** Receptor soil, **(B)** Donor soils.

The receptor soil (R, that receive the soil donation) and donor soils (D, which serve as source of the desirable microbiota), were taken from the top 0–20 cm depth excluding roots (bulk soil). The R soil was a Andisol from Barros Arana series (**Figure 1A**) and five Antarctic D soils were collected from the Antarctic campaign ECA-55 (**Figure 1B**): (Y) Yelcho station, (C) Coppermine Peninsula, (A) Arctowski station, (F) Fildes Bay and (D) Deception Island. Total nitrogen (N), phosphorus (P), potassium (K), sodium (Na), calcium (Ca), magnesium (Mg), aluminum (Al), Effective Cation Exchange Capacity (ECEC), pH, and soil organic matter (SOM) were determined as described in Sadzawka et al., 2006 and water-holding capacity (WHC) were calculated using a cylinder sand bath method (Brischke and Wegener, 2019).

To conduct the *in-situ* microbiota selection experiment, 20% of each type of D soil was transferred to R soil (80%) (Durán et al., 2017). Tomato seeds (cv. Cal Ace) were planted in pots containing 200 g of the soil mixtures. The plants were cultivated in a 16/8 h day/night cycle at temperatures of 21/15°C, 70% relative humidity, and photosynthetic photon levels of 1,100-500 µmol m^-2^s^-1^ for a period of 4 weeks. The Taylor and Foy nutrient solution was applied every 15 days (Taylor and Foyd, 1985). These conditions were maintained for a 4-week period. Subsequently, the aerial parts of the seedlings were harvested, while the roots were left in the soil.

**Figure 2:**
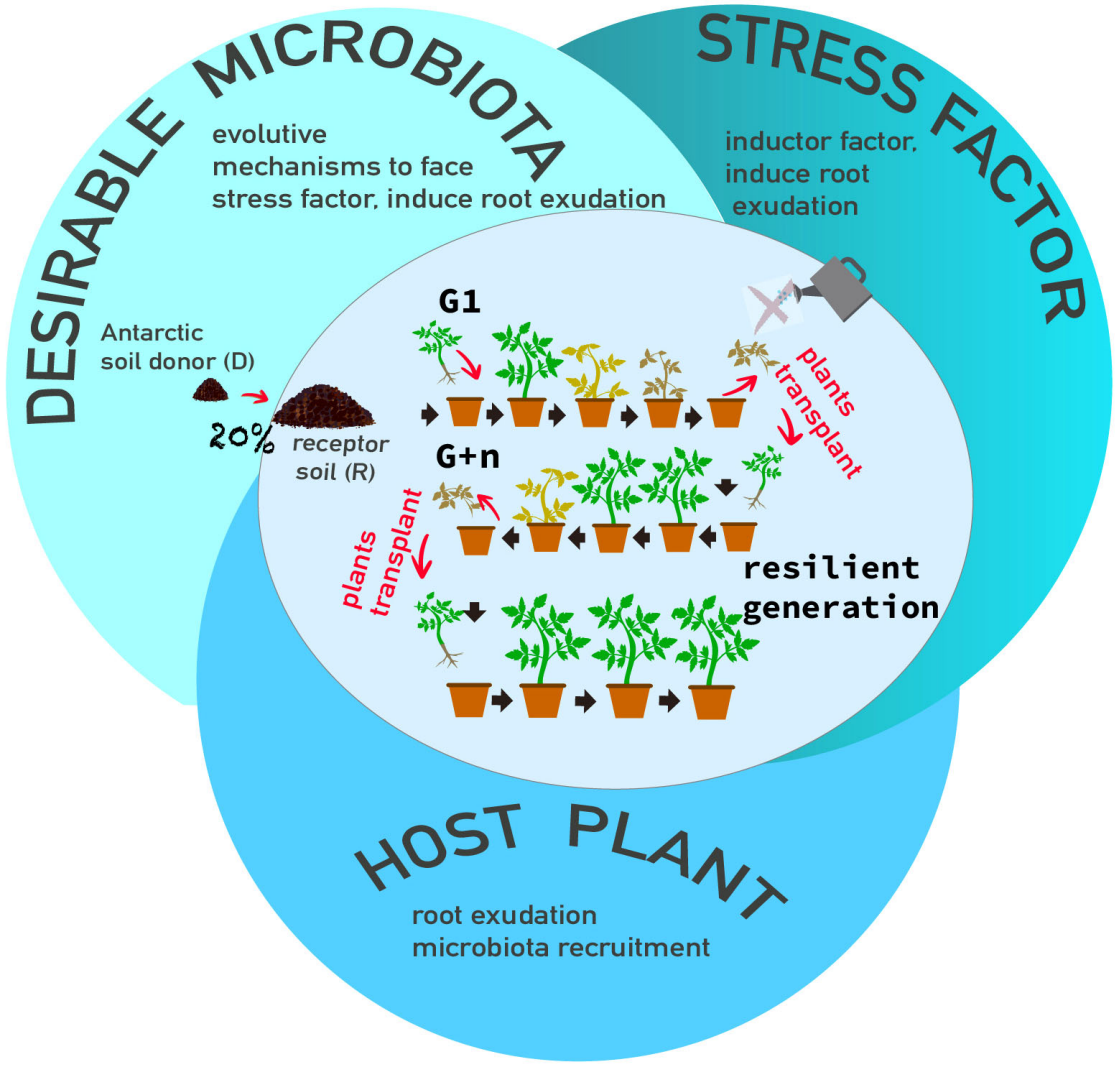
Recruitment of *in situ* microbiota through tridimensional factors: i)A desirable microbiota as microorganisms donor, ii)a stressor factor as inducer, iii)a host model as modulator. Multigenerational selection of microbiomes is shown in the figure, tomato plants were stressed with water deficit, cut, and new plantlets (same cultivar) were transplanted in the same soil and the cycle is repeat until the plants remain without water deficit symptoms (resilient generation).

This process corresponds to generation 0 (G0) of the HMME approach, which involves planting new seedlings (of the same cultivar) into the soil from the previous generation with the aim of enhancing the host’s ability to modulate its associated microbiome (**Figure 2**). To establish multigenerational selection, seeds from the same cultivar were sown in the same soil as G0, marking the beginning of G1 in the HMME approach. The pots containing each soil combination and the R control soil were divided into two groups: i) water deficit stress treatment, and ii) well-watered control. The seedlings were kept under the same conditions described for G0, with the exception of varying irrigation regimes. Water deficit stress was induced after 20 days of establishment, during which the tomato seedlings were irrigated to reach 90% of Water Holding Capacity (WHC). Subsequently, irrigation was reduced to only 50% of WHC until the seedlings exhibited symptoms of reduced photosynthetic pigments corresponding to Matrix Scale-Based (MSB) stage 4 (as described below). The seedlings were then pruned, and the process was repeated to initiate a new cycle (new generation).

In this study, a total of 10 generations (10 cycles) were carried out. This 50% WHC treatment was applied from generation 1 (G1) through generation 3 (G3). As the generations progressed and the plants exhibited increased tolerance to water deficit, irrigation was further decreased by an additional 10%, ensuring clear evidence of water deficit stress symptoms. Consequently, irrigation was reduced to 40% of WHC in G4-G5, 20% in G6, 10% in G7, and a mere 5% in G8-G10. Control plants were consistently maintained well-irrigated (at 90% of WHC) throughout all generations. The entire study was conducted over a span of 96 weeks.

With the aim of assessing stress levels, a dedicated stress index was conceived and developed for this study, in order to establish and standardize the sampling moment. Symptom incidence was registered using a “Matrix Scale-Based (MSB)” assignment, with numeric values from 1 to 6 based on percent of leaf area showing chlorosis at 1 (0-10%), 2- (11-30%), 3- (31-50%), 4- (51-70%), 5- (71-90%) and 6- (90-100%) (**Supplementary Figure 1** and **Supplementary Equation 1**).

Samples (leaf, root, and rhizosphere) were collected at initial state before multigenerational selection (G0), low tolerance (G2), moderate tolerance (G5) and high tolerance to water deficit (G10). Leaf proline, an amino acid overproduced was quantified according Carillo and Gibon, 2011. Briefly, 0.05 g of leaves were macerated with 1.5 mL of 40:60 ethanol: water. The extract was incubated at 4°C for 24 h and then centrifuged at 10,000g x 5 min. After centrifuging, 50 µL of the supernatant was mixed with 100 µL of ninhydrin 1% (w/v), acetic acid 60% (v/v) and ethanol 20% (v/v). The reaction mixture was incubated for 20 min at 95°C and then chilled on ice for 5 min. Finally, the sample was read at 520 nm. For the calibration curve, 0 to 0.3 mM of proline standard was used. Rhizosphere soils from each generation were stored for subsequent metabarcoding analyses.

### 2.2 Taxonomic diversity and functional annotation of prokaryotic taxa by metabarcoding analyses

Total genomic DNA from rhizosphere samples were extracted using MO BIO PowerSoil DNA Isolation Kit (Qiagen). The V3-V4 regions of 16S rRNA gene were amplified using primers Bakt341F (5’-CCTAYGGGRBGCASCAG-3’) and Bakt806R (5’-GGACTACNNGGGTATCTAAT-3’). Amplicons were purified and dual indices and sequencing adapters were attached to each sample using the Nextera XT Index Kit (Illumina, San Diego, California, U.S.A.). Equimolar concentrations of samples were sequenced for 500 cycles in paired-end mode (250x2) on the Illumina NovaSeq 6000 instrument (Illumina, Inc., San Diego, CA, USA). Resulting reads were processed using QIIME2 (Quantitative Insights into Microbial Ecology) software package (Bokulich et al., 2018), where an error correction model was trained using DADA2, resulting in biologically relevant sequences known as Amplicon Sequence Variants (ASVs) (Callahan et al., 2016). The taxonomy of ASV’s were assigned using the SILVA138 database. The samples were rarified at a depth of 68915 amplicons, and these abundances were used as input for measurements of diversity (Shannon’s diversity index and Faith’s Phylogenetic Diversity) and richness (Features) using QIIME2. A mixed-effect model was built for every alpha diversity index, to evaluate the fixed effects of generation, treatment, and soil mixture, considering the random effect related to every sample id, these models were built using *lmer* function of lme4 R package, and the variance partitioned with *r2beta* function of r2glmm R package. Beta diversity was explored using a principal component analysis (PCA) on Aitchison distances, and the statistical determination of community differences between treatments were evaluated using permutational multivariate analysis of variance (PERMANOVA,(Anderson, 2008)) with 999 random permutations with the *adonis* functions from the *vegan* package (Son et al., 2007) contained in R environment. The data was standardized, centered, and scaled prior to performing PCA. The raw counts abundances were normalized using a cumulative sum scaling (CSS) approach at genus level, and were tested between control and treatment in G10 of every soil mixture using a Zero Inflated Gaussian Mixture Model (ZIGMM), implemented in metagenomeSeq R package (Paulson et al., 2013). Finally, to explore the functional profile of the samples, the identified ASVs at least at family taxonomy level, were queried to the Functional Annotation of Prokaryotic taxa (FAPROTAX) database, assigning environmental functions (Louca et al., 2016).

### 2.3 Validation of the role of transferred microbiome in water deficit tolerance

To diminish the microbial load and corroborate the role of the microorganisms in plant fitness fumigations using chloroform (CHCl_3_) from resilient generation (G10) soils was performed. Briefly, 30 g of soil were fumigated in a vacuum desiccator with 200 mL of CHCl_3_ for 24 h at room temperature (Jenkinson and Powlson, 1976). In parallel, no-fumigated vacuum desiccator without chloroform was incubated. After CHCl3-fumigation, tomato seeds (same cultivar) were sown in a fumigated and no-fumigated soil and were growing in a greenhouse during the day/night cycle of 16/8 h, 21/15°C, 70% relative humidity and 90% WHC. After that, serial dilutions of soil extracts were performed with LB agar media to determine the cultivable microbial load. Finally, tomato seeds were grown at 50% WHC after acclimatation. The stress level was determined and proline concentration was quantified.

### 2.4 Statistical analyses

Proline content and chemical analysis of soils were analyzed by ANOVA and with a *post hoc* Tukey’s multiple pairwise comparison using SPSS V.25 software package (SPSS, Inc.). Values were given as the means ± standard errors. To identify significant changes in chemical properties after making the mixtures, a comparison of two means was made between the donor soils and soils that contained the transferred microbiome at the P<0.05 probability level according to the student’s t test.

## 3 Results

### 3.1 Tolerance of tomato plants to water deficit

This study examined the impact of HMME on tomato plants subjected to water deficit. Donor soils (Y, C, A, F and D), were used to transfer desirable microbiota to receptor soil (R), where tomato seeds were sown. Taxonomic diversity and functional annotation of prokaryotic taxa were analyzed, and the role of the transferred microbiome in water deficit tolerance was validated.

In general, plants subjected to water deficit showed significantly less biomass compared with control well-watered plants. However, R soil transplanted with soil from Fildes Bay showed no-significant differences in biomass in all generations when compared to the control (**Supplementary Figure 1A**). Leaf proline values in water-stressed tomato leaves tended to decrease as the generations advanced. In fact, in G10, in general no significant differences in proline production were observed for water deficit treatments as compared to the well-watered control (**Supplementary Figure 1B**). Whereas, in G2 and G5, the tomato leaf proline levels under water deficit stress were 3.0-4.5 and 1.5-2.5 times higher, respectively, than those of the well-watered seedlings. This behavior was also demonstrated in plants grown in control R. This is supported by evidence based on the Matrix Scale Based MSB scale, where plants challenged with water deficit had fewer symptoms and enhanced survival rate after multiple generations of stress exposure. Indeed, G1 and G2 plants survived 4 weeks, and G3, 7 weeks at 50% WHC. The G4 and G5 plants survived 7 and 8 weeks at 40% WHC and G6 survived 5 weeks, but at 20% WHC. In G7, irrigation was supplied at 10% WHC and plants survived for 4 weeks. Finally, G8 and G9 plants survived 4 weeks, and G10, 5 weeks at only 5% WHC (**Supplementary Figure 2**). This also was confirmed by the stress index, which considers all factors, and revealed major tolerance in the last generations (G8, G9 and G10), particularly for seedlings grown in R+C, R+D, and R+F soils at G10 (**Supplementary Figure 3**).

### 3.2 Community composition and functional prediction of rhizosphere microbiota in response to water deficit

**Figure 3:**
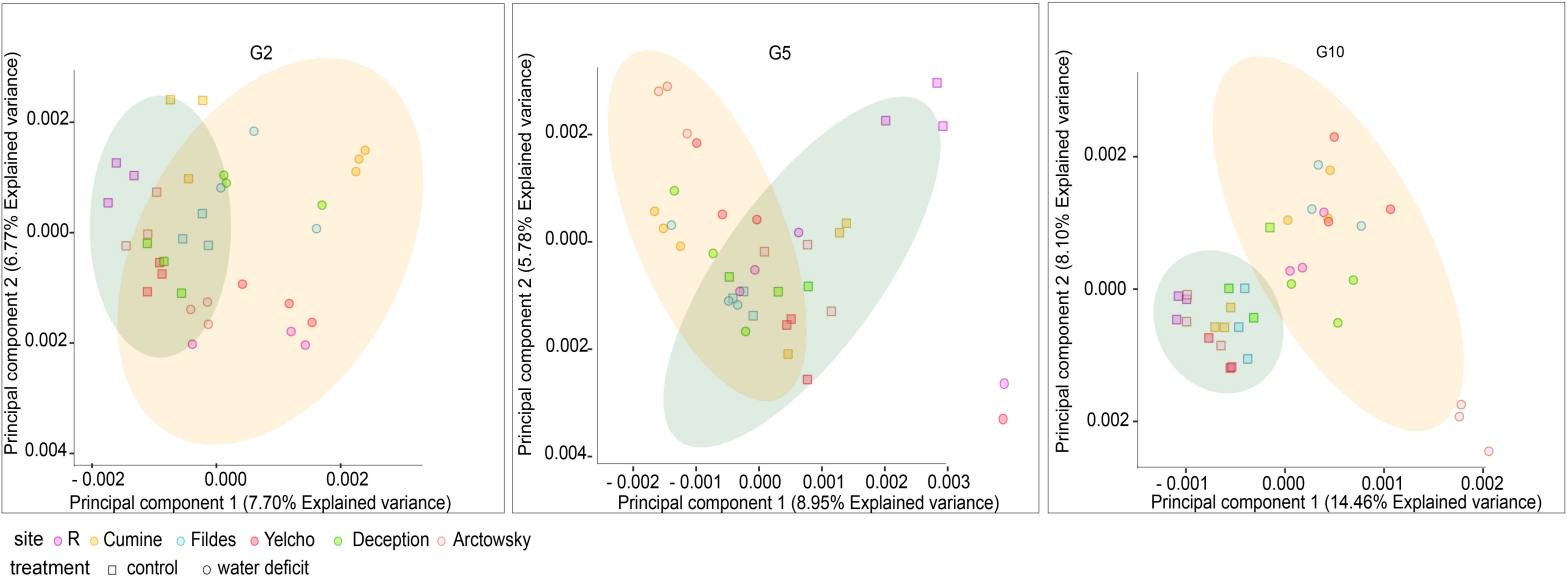
Principal component analyses based on relative abundance results of metabarcoding results of well-watered and water deficit treatments in G2, G5 and G10. G= generation, R= Receptor soil, R+C (receptor mixed with Coppermine Antarctic soil); R+D (receptor mixed with Deception Antarctic soil); R+F (receptor mixed with Fildes Antarctic soil); R+Y (receptor mixed with Yelcho Antarctic soil); R+A (receptor mixed with Arctowsky Antarctic soil).

A clear differentiation in microbial communities between water deficit treatment and well-watered control was observed as the generations progressed (**Figure 3**; **Supplementary Table 2**). Water deficit as a variable better explained the statistical variation among soil microbial communities, explaining 6.8% of community variance in G2 and 11.6% in G10. In general, the Shannon index decreased in all the soil mixtures as the generations progressed, with generation variable explaining 71.9% of community variances in diversity. Faith’s phylogenetic diversity values decreased across generations only in the control samples, with the ‘treatment’ variable explaining 12.6% of community diversity variance. Finally, Pielou’s evenness index also decreases throughout the generations, which explained 64.6% of the modeled variance in evenness (**Supplementary Figure 4**).

The relative abundances of the rhizobacterial taxa at phylum and genus levels shifted across the generations G0, G2, G5 and G10 in both control and water deficit treatments as shown in **Supplementary Figures 5**–**7**. Members of the Proteobacteria were relatively the most abundant taxa in all soils of G0, (34-38%), followed by Actinobacteria (19-27%). Acidobacteria was the third most abundant phylum in all soil samples, however, its relative abundance decreased until reaching G10. In well-watered controls, in the G2, R, R+D and R+A relative abundances of Actinobacteria increased from 34 to 37%, and became more dominant than Proteobacteria (25-36%) in these soils. Similarly, in water deficit conditions, Firmicutes increased in all treatments compared to G0. In G5, Actinobacteria increased and Proteobacteria abundances declined in all soils except in R+F. Similar to G2, in G5 Firmicutes decreased in all treatments compared to their corresponding G0 compositions. In G10, Proteobacteria increased in relative abundances in the rhizosphere of all well-watered controls and in also in the R, R+C and R+D rhizosphere soils under water deficit conditions. Firmicutes increased in all treatments subjected to water deficit. Also, all treatments exposed to water deficit had increased relative abundances of the archaeal phylum Thaumarchaeota, which was particularly notable in R+F soils, where the relative abundances increased from 0.45% to 9.44% between G0 and G10.

**Figure 4:**
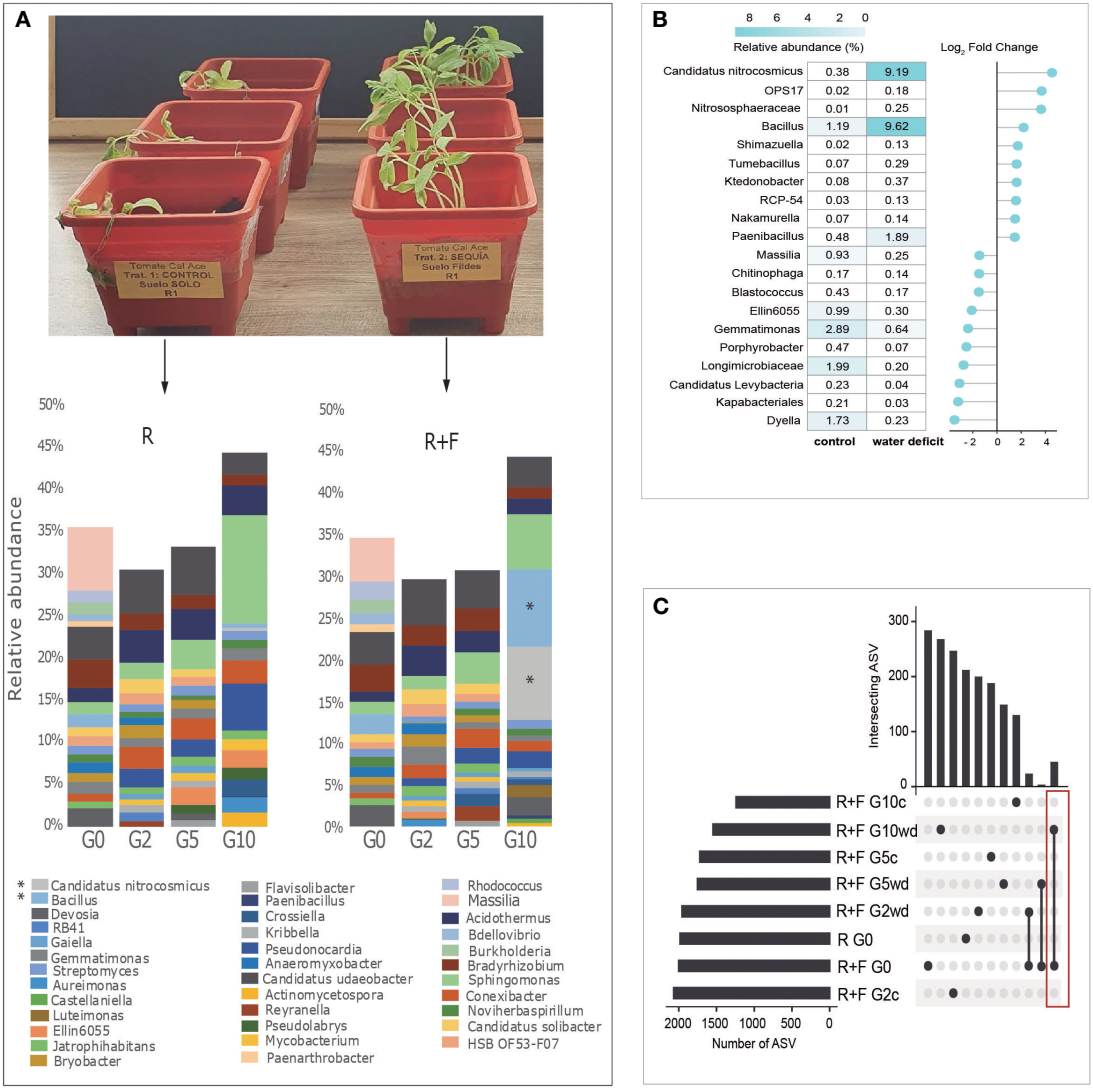
**(A)** Taxonomic diversity by metabarcoding analyses of 16S RNA gene of Receptor soil (R) and Receptor soil+Fildes bay (R+F) at generation G0, G2, G5 and G10 (G= generation). **(B)** Differential abundance by ZIGMM using metabarcoding data in generation 10 of R+F treatment. Negative values in Log_2_ Fold change represent a loss in the relative abundance of the species and positive values in Log_2_ Fold Change represent gains in relative abundance for each taxonomic group. **(C)** Upset analysis of metabarcoding analysis of the region V3-V4 16s rRNA gene of the rhizosphere soil of tomato plants grown in Fildes soil (F) mixed with a Andisol receptor soil (R). The plants were well-watered **(C)** or subjected to water deficit (WD) and subjected to host-mediated microbiome engineering. Metagenomic analysis were performed after generation 2 (G2), generation 5 (G5) and generation 10. Red rectangle indicates the ASV that remain for 2 years (i.e., 45 ASV) along the generations exposed to water deficit.

*Candidatus* Nitrosocosmicus and *Bacillus* spp. were the most abundant taxa after G10 in the R+F soil (**Figure 4A**), i.e., in tomato plants that reached a greater water stress tolerance. ZyGMM analysis of R+F treatment revealed differential abundances of *Candidatus* Nitrosocosmicus, which was 9.19% of the community in water deficit treatments and 0.38% in well-watered control samples and had the highest Log_2_ Fold change of all taxa (**Figure 4B**). Also, *Bacillus* comprised 9.62% of the community in the water deficit treatment and 1.19% in the well-watered control samples. The most abundant and ubiquitous genus of the Proteobacteria phylum was *Sphingomonas* and the most common Actinobacteria was *Acidothermus*, which were increased across the generations, but no significant differences were found between well-watered control and water deficit treatments (**Supplementary Figure 5**).

Interestingly, several bacterial strains coming from Antarctic soils remained into the rhizophore microbiota of the soil mixtures after 96 weeks. For example, 45 taxonomic groups (ASVs observed) from Fildes Bay soil were maintained throughout the multigenerational selection process under water deficit conditions (**Figure 4C**). Results from differential abundance by ZyGMM analysis of all soil mixtures in G10 are shown in **Supplementary Figures 9**–**13**.

**Figure 5:**
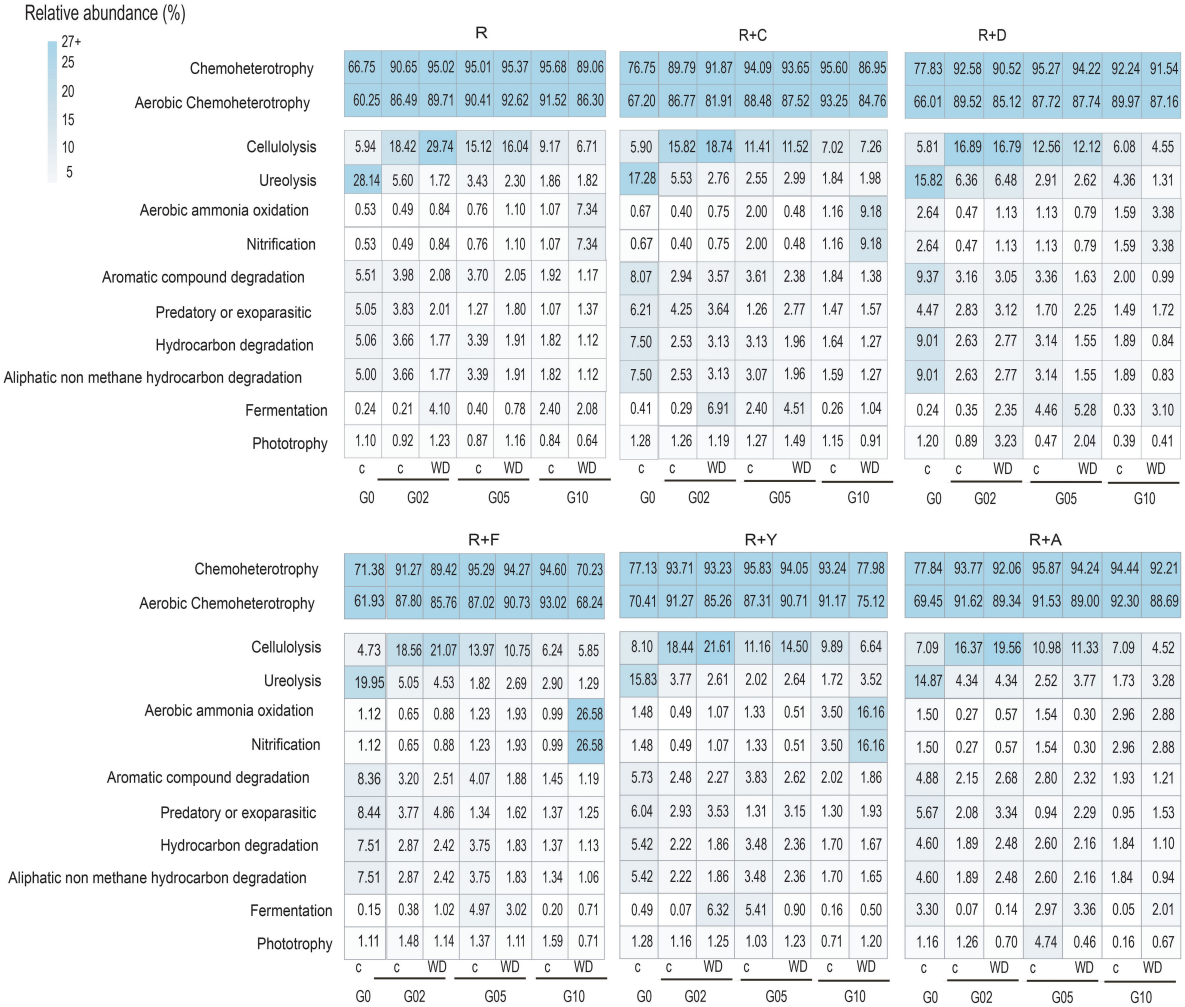
Functional Annotation of Prokaryotic Taxa analysis of R+F treatment of all generations. G= generation, R= Receptor soil, R+C (receptor mixed with Coppermine Antarctic soil); R+D (receptor mixed with Deception Antarctic soil); R+F (receptor mixed with Fildes Antarctic soil); R+Y (receptor mixed with Yelcho Antarctic soil); R+A (receptor mixed with Arctowsky Antarctic soil). C=control; wd= water deficit.

The functional annotation of the metabarcoding data revealed putative changes in several microbiota functional potentials as the experiment progressed. Chemoheterotrophic and aerobic chemoheterotrophic were identified as the dominant functions in all treatments and generations, comprising over 60% of all potentials in each treatment. Additionally, putative variations were observed in processes related to the nitrogen cycle across generations. Specifically, the potential for aerobic ammonium oxidation showed a progressive increase with successive generations, especially in the R+F treatments (**Figure 5**). On the other hand, functions related to ureolysis notably decreased as generations advanced. Methanogenesis, including hydrogenotrophic methanogenesis and methanogenesis through the reduction of methyl compounds with H_2_, also declined as generations progressed, both in control soils and under water deficit treatments. The putative results of FAPROTAX for all soil mixtures are shown in **Figure 5**.

### 3.3 Chemical soil parameters after soil donation

To comprehend alterations in the chemical properties of soils prior to and post mixing, chemical analyses were conducted (**Supplementary Table 1**). Generally, the donor soils exhibited low nitrogen content and high available phosphorus content in comparison to soil R. For potassium, the donor soils showed values ranging from 100 to 260 mg kg^-1^, while soil R had 130 mg kg^-1^. pH values within the donor soils displayed a wide range, spanning from 4.36 to 8.29. Organic matter content in the donor soils was lower (ranging from 1.08% to 1.70%) compared to soil R (13.23%), except for soil Y (22.33%). In terms of effective cation exchange capacity (ECEC), donor soils generally exhibited higher exchangeable cation content compared to soil R. Soil Y contained notably higher aluminum content (2 cmol^+^ kg^-1^) and a greater aluminum saturation percentage (41.46%) compared to the other soils.

Values obtained for macronutrients (N, P, and K) in the mixed soils tended to align closely with those in soil R (P > 0.05). However, soils R+C and R+F demonstrated significantly lower nitrogen content (60% and 24% lower, respectively) compared to soil R. Soil R+Y displayed notably higher phosphorus content (37 mg kg^-1^) than soil R (2 mg kg^-1^), whereas the mixtures R+F and R+A exhibited significantly lower potassium content (99 and 74 mg kg^-1^, respectively) than soil R (131 mg kg^-1^). Concerning pH, only soil R+Y (pH = 5.31) displayed a significant difference. Soil mixing did not yield significant pH differences in any of the utilized soils. Organic matter content showed similarities across all soil mixtures except for the R+Y mixture (16.77%). Noteworthy disparities in exchangeable cations were observed across all samples. Water holding capacity (WHC) values ranged from 66% to 82%, and all mixtures, except R+C, exhibited significantly different WHC values compared to soil R.

**Figure 6:**
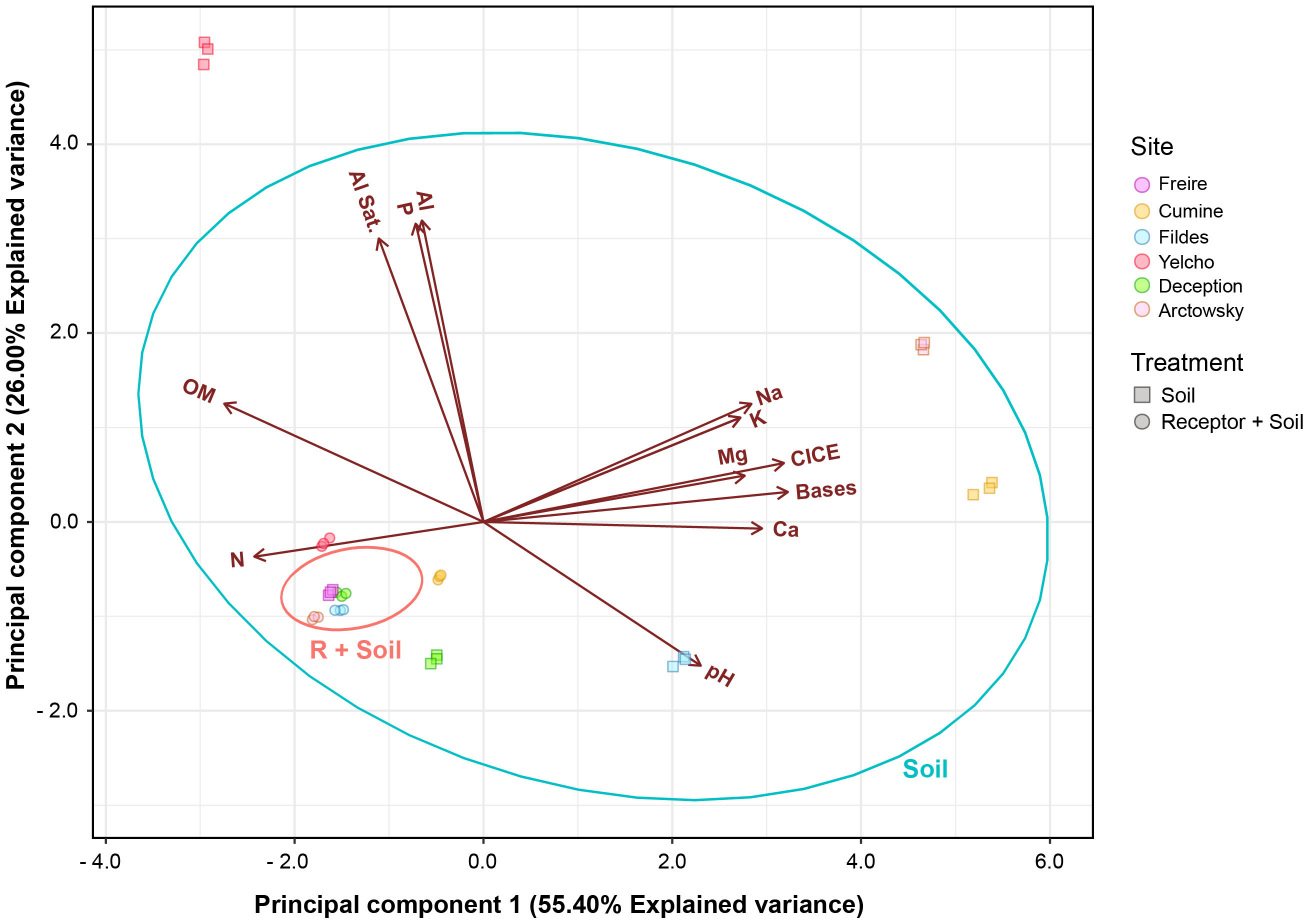
Principal component analysis of Chemical parameters of all mixed and unmixed soils before the experiment.

Similarities in the chemical soil characteristics before soil donation were observed, with the R+Y treatment being the only one showing significant differences (**Figure 6**). These differences could be attributed to higher P content (37 mg kg^-1^), pH, and organic matter, which were statistically distinct from the other soils. Soil macronutrients (N, P, and K) in the mixed soils exhibited similarities to the receptor Andisol (P > 0.05). Notably, significant differences were noted in the exchangeable cations across all samples. Water holding capacity (WHC) values ranged from 66 to 82%, and all mixes except R+C displayed significantly different WHC compared to the R soil.

### 3.4 Validation of role of transferred microbiome

Soil chloroform fumigation assay was performed to elucidate the influence of microorganisms on the water deficit stress tolerance of tomato plants. Our results showed a reduction of cultivable bacteria after chloroform fumigation (F+) by at least 64-70% (**Supplementary Figure 8A**). This decrease in microbial load produced a two-fold increase in plant stress index compared to plants in the non-fumigated (F-) soil (**Supplementary Figure 8B**). Similarly, proline content was more than tenfold in plants in F+ soil compared to those in F- (from 3 to 40 µmol g^-1^ FW) (**Supplementary Figure 8C**).

## 4 Discussion

The potential for microbiome soil transplant to improve tomato plant tolerance against water deficit stress was assayed through the HMME after Antarctic microbiome soil donation. The Antarctic soil microbiomes were chosen as sources of desirable microorganisms due to their inherent capability to withstand extreme arid conditions characterized by limited liquid water availability (Morales-Quintana et al., 2021; Ball et al., 2022).

A noticeable enhancement in the tolerance of tomato seedlings to water deficit stress was observed in all soil mixtures throughout successive generations of HMME. This was evidenced by a significant improvement in survival time, plant stress index decrease, biomass accumulation, and a reduction in leaf proline content. This enhanced plant response was primarily evidenced in R+F, which had the highest plant biomass under water deficit in G10. In general, at the beginning of the study, higher levels of proline were found in leaves of tomato seedlings subjected to water deficit as compared to the well-irrigated control. The proline levels gradually decreased in the leaves of plants grown in each soil mixture over successive generations, with a more pronounced decline becoming apparent from G5 onwards. Concurrently, an evident improvement in tolerance to water deficit stress began to manifest. The intracellular accumulation of osmolytes, such as proline, represents a crucial physiological plant response aimed at mitigating cell membrane damage and preserving cellular integrity (Claussen, 2005; Montesinos-Pereira et al., 2014). Elevated proline levels have been linked to the response to water stress, which can be also induced by inoculating the plant with PGPB. (Wang et al., 2012; Palika et al., 2013; Shintu and Jayaram, 2015). For example, *Bacillus* spp. have been shown to be efficient PGPB that also have the capability to reduce proline levels in plants (Wang et al., 2012; Palika et al., 2013; Shintu and Jayaram, 2015; Mehboob et al., 2022). The decreased levels of proline, together with the improved performance of the tomato seedlings under water deficit, which were even comparable to the well-irrigated controls, indicated that the plant ceased to be dramatically stressed towards the last generations of the study. While undergoing generations, the rhizosphere microbiota associated with tomato plants was concurrently restructured and microbial alpha/beta diversity indices declined across generations. Thus, microbial communities became phylogenetically more similar and taxa abundances were more uniform within treatments, but communities became more dissimilar from one another as generations progressed. The most dominant phyla found in the rhizosphere soil of tomato seedlings in all soils over all generations were *Proteobacteria*, *Actinobacteria*, *Acidobacteria* and *Firmicutes*. These phyla consistently emerge as dominant taxa in the rhizosphere soils of diverse plant species (Jochum et al., 2019; Mueller et al., 2019; Jiang et al., 2022), including those inhabiting Antarctic soils (Zhang et al., 2020) and Andisols (Lagos et al., 2014; Acuña et al., 2020). Possible reasons for their prevalence lies in these bacterial taxa possessing specific life strategies and characteristics that enable them to endure various environmental stresses. These include fast growth rates (Ling et al., 2017), metabolic versatility (Lladó and Baldrian, 2017) and phenotypic plasticity (Perraud et al., 2020). For example, the spore-forming capacity of *Firmicutes* and *Actinobacteria* allows them to enter a dormant state during periods of stress by water deficit, while the less suitable bacterial lineages decreases in abundance (Naylor et al., 2017). Although the dominant taxa persisted, water deficit and multigenerational selection significantly influenced on the rhizobacterial microbiota composition, which could be linked with changes in soil moisture and the release of primary and secondary metabolites by tomato plants (Bakker et al., 2018; Berendsen et al., 2018b; Li et al., 2019; Rolfe et al., 2019). These findings align with the hologenome theory of evolution, which postulates that eukaryotic organisms establish close relationship with microbiota that could be inherited and exert significant effects on host fitness (Carrier and Reitzel, 2018). The host-associated microbiota can undergo dynamic changes, influenced by modifications in the environment, host factors, and microbial genomes (Carrier and Reitzel, 2018). We unveiled a notable variation in the structure of the bacterial microbiota across each evaluated generation, highlighting the dynamic nature of these host-microorganism interactions. As expected, the rhizospheric microbiota underwent an adaptation process in intermediate generations (G2 and G5), where several taxonomic taxa as *Rhodococcus* and *Paenibacillus* were suppressed, while *Sphingomonas* was enhanced. Overall, when assessing the multigenerational-selected soil microbiome (after G10) under both control and water deficit conditions, the bacterial genera *Sphingomonas* and *Bacillus*, along with the archaea *Candidatus* Nitrosocosmicus, emerged as the most prevalent taxa. Notably, under water deficit stress conditions, *Bacillus* and *Candidatus* Nitrosocosmicus exhibited higher abundance compared to other taxa, reflecting the impact of the HMME approach on shaping the microbial composition. *Bacillus* spp, are ubiquitous inhabitant of soils, with a wide distribution in natural environments and are frequently associated with diverse plant species and niches. Numerous publications have evidenced the importance of species from this genus in alleviating water deficit stress in plants *via* phytohormone synthesis, volatile compounds production, increasing nutrient availability and the activity of the enzyme ACCD, which reduces ethylene-induced stunting in plants (Barra et al., 2016; Durán et al., 2016; Gagné-Bourque et al., 2016; Barra et al., 2017; Tiwari et al., 2018; Moreno-Galván et al., 2020; Astorga-Eló et al., 2021). The increase in abundance of *Candidatus* Nitrosocosmicus (Archaea, Thaumarchaeota phylum) was evidenced in all soils after G10, but especially in tolerant plants to water deficit from R+F soils. Members of this phylum are relatively rare across many environments but seem to be particularly abundant in desert soils (Fierer et al., 2012; Marusenko et al., 2013). This archaea has been reported to be involved in biogeochemical cycles, particularly in carbon and in nitrogen cycles, as an ammonium oxidizing archaea (AOA), important for the control of soil nitrification (Lehtovirta-Morley et al., 2016; Wang et al., 2019). A previous work described that this archaea is tolerant to prolonged water deficit events (Hammerl et al., 2019), therefore water deficit would benefit its proliferation with respect to other less tolerant microorganisms. Due to the remarkable increase in abundance of *Bacillus* spp. and *Candidatus* Nitrosocosmicus under water deficit and their consistent dominance suggests their potential involvement in key functional processes within the soil ecosystem, particularly under water deficit stress conditions. In addition, it is well-documented that chloroform treatment can significantly reduce the microbial population size by at least 70% (Ingham and Horton, 1987). Therefore, to further validate the role of the microbiome in water stress tolerance, fumigation of soils was carried out after G10, after which new seedlings were grown in the fumigated soils. After fumigation, plants once again became susceptible to water deficit stress, strongly indicating the significant role of the microbiota in conferring acquired tolerance to water deficit in plants. It is also important to highlight that given the inherent soil buffering capacity, the transplantation of donor soils had minimal effects on key soil receptor attributes, including macronutrient levels, soil organic matter (SOM) content, and pH values (Nanzyo, 2002; Ugolini and Dahlgren, 2002). Therefore, we propose that these prokaryotic taxa could play a significant role in enhancing the tolerance of tomato seedlings to water deficit, especially in soils engineered with soils from Fildes Bay. Although there is a noticeable trend of enrichment with these taxa in the engineered soils associated with enhanced water stress tolerance, further research is needed to provide stronger evidence and confirm these findings. Additional studies are required to investigate the molecular and physiological basis of their interactions with plants, as well as their potential roles in nutrient acquisition and stress mitigation pathways. Moreover, it is essential to unravel the specific mechanisms and interactions by which these taxa could contribute to plant-microbe associations and nutrient cycling processes. By comprehending the functional contributions of *Bacillus* and *Candidatus* Nitrosocosmicus, we can advance the fine-tuning and optimization of the microbiome engineering approach for the development of sustainable and efficient biofertilization strategies.

The beneficial role of plant-associated microorganisms isolated from the rhizosphere and utilized as plant bioinoculants under conditions of water deficit stress has been extensively demonstrated, providing promising results under controlled experimental conditions (Asaf et al., 2017; Ximénez-Embún et al., 2018; Zhang et al., 2018; Kim et al., 2022). However, studies investigating the impact of environmental stressors, such as water deficit stress, on the adaptation, composition and functionality of plant-associated microbiota over time are relatively recent and limited in number. In this context, the FAPROTAX analysis is a valuable and accurate tool utilized for rapid functional screening and grouping of data derived from the 16S rRNA gene in terrestrial ecosystems (Sansupa et al., 2021). As expected, chemoheterotrophy and aerobic chemoheterotrophy were the predominant functions in all samples and treatments, possibly due to the environment in which the plant developed. However, the significant increase in the relative abundance of AOA, particularly *Candidatus* Nitrosocosmicus, particularly in the R+F treatment under water deficit conditions suggests a potential link between enhanced water deficit tolerance and increased oxidation of ammonium (NH_4_^+^) or ammonia (NH_3_) to nitrate (NO_3_^-^) in the soil. This, in turn, may lead to an augmented nitrogen absorption capacity by the plant. Nevertheless, studies of the functionality of the plant-associated microbiota, including transcriptomic and proteomic analysis, throughout and after the HMME approach are still necessary to corroborate this hypothesis.

## 5 Conclusions

Our findings demonstrate the potential to enhance the water deficit tolerance of tomato plants by transferring the Antarctic microbiome through 20% w/w soil inoculation. This enhancement could be achieved through the recruitment, maintenance, and restructuring of microbiomes *via* multigenerational plant selection. Interestingly, our results show that specific taxa from Antarctic soils were capable of persisting in the tomato rhizosphere throughout the entire 96-week experiment. For instance, 45 taxonomic groups (ASVs observed) from Fildes Bay remained consistent during the multigenerational selection process under water deficit conditions. This suggests that some taxonomic groups co-evolved with the host plant during the multigenerational water deficit selection.

When examining the relative abundance data across the course of multigenerational selection, it becomes evident that *Bacillus* spp. and *Candidatus* Nitrosocosmicus could potentially serve as pivotal contributors to enhancing water deficit tolerance in tomato seedlings, particularly in soils from Bahía Fildes. However, further trials are needed to validate this hypothesis. It is worth noting that understanding the underlying mechanisms governing microorganism-plant interactions is vital for optimizing crop productivity in a rapidly changing world. For instance, exploring the organic exudates utilized during host signaling in the ‘cry for help’ process, studying interactions within the plant endosphere, and unraveling the assembly mechanisms of beneficial microbiota can pave the way for the development of more resilient bioinoculants suitable for agroecosystems.

## Data availability statement

The original contributions presented in the study are included in the article/**Supplementary files**, further inquiries can be directed to the corresponding author/s.

## Author contributions

RR, PB and PD wrote the main manuscript text. GL analyzed the metagenomic data. PB, VC, PD, and LH critically revised the manuscript and approved the final version. All authors contributed to the article and approved the submitted version.

## Supplementary material

Supplementary Figure 1
**(A)** Dry matter of shoot and root of tomato plants and **(B)** Proline content in shoot of tomato plants. G= generation, R= Receptor soil, R+C (receptor mixed with Coppermine Antarctic soil); R+D (receptor mixed with Deception Antarctic soil); R+F (receptor mixed with Fildes Antarctic soil); R+Y (receptor mixed with Yelcho Antarctic soil); R+A (receptor mixed with Arctowsky Antarctic soil).

Supplementary Figure 2 Matrix based scale (MSB) of tomato plants exposed to water deficit stress based to Water-Holding Capacity (WHC) and visualized according to chlorosis scale. From G1 to G3 the water deficit treatments were irrigated at 50% WHC. from G4 to G5 the irrigation decreased to 40% WHC. The G6 was irrigated at 20% WHC and G7 was irrigated at 10% WHC. Finally, from G8 to G10 the plants were irrigated at 5% WHC. G=Generation.

Supplementary Figure 3 Stress index obtained from the MSB taking account the equation 1.

Supplementary Figure 4 Alpha diversity of all soil samples**. (A)** Shannon’s diversity index, **(B)** Pielou´s evenness, **(C)** Features (richness) and **(D)** Faith’s Phylogenetic Diversity of all data and all generations.

Supplementary Figure 5 Relative abundance of each taxa at the phylum level in the total rhizobacterial community associated with G2, G5 and G10 in water deficit and control treatments. G=generation.

Supplementary Figure 6 Relative abundance of each taxa at the genus level in the total rhizobacterial community associated with G2, G5 and G10 in control treatments. G=generation.

Supplementary Figure 7 Relative abundance of each taxa at the genus level in the total rhizobacterial community associated with G2, G5 and G10 in water deficit treatments. G= generation

Supplementary Figure 8 Unit formed of colony **(A)**, stress index obtained from the MSB taking account the equation 1, G= generation **(B)** and Proline content on fumigated (F+) and not fumigated (F-) treatments with chloroform.

Supplementary Figure 9 Differential abundance by ZIGMM using metabarcoding data in G10 of R treatment. Negative values in Log_2_ Fold change represent a loss in the relative abundance of the species and positive values in Log_2_ Fold Change represent gains in relative abundance for each taxonomic group.

Supplementary Figure 10 Differential abundance by ZIGMM using metabarcoding data in G10 of R+A treatment. Negative values in Log_2_ Fold change represent a loss in the relative abundance of the species and positive values in Log_2_ Fold Change represent gains in relative abundance for each taxonomic group.

Supplementary Figure 11 Differential abundance by ZIGMM using metabarcoding data in G10 of R+C treatment. Negative values in Log_2_ Fold change represent a loss in the relative abundance of the species and positive values in Log_2_ Fold Change represent gains in relative abundance for each taxonomic group.

Supplementary Figure 12 Differential abundance by ZIGMM using metabarcoding data in G10 of R+D treatment. Negative values in Log_2_ Fold change represent a loss in the relative abundance of the species and positive values in Log_2_ Fold Change represent gains in relative abundance for each taxonomic group.

Supplementary Figure 13 Differential abundance by ZIGMM using metabarcoding data in G10 of R+Y treatment. Negative values in Log_2_ Fold change represent a loss in the relative abundance of the species and positive values in Log_2_ Fold Change represent gains in relative abundance for each taxonomic group.
