# Supplementary material for: Microbiome engineering optimized by Antarctic microbiota to support a plant host under water deficit
Source: Front Plant Sci. 2023 Sep 15;14:1241612. doi: 10.3389/fpls.2023.1241612 (PMC10541027; doi:10.3389/fpls.2023.1241612)
Supplement: Supplementary Figure 1 — (A) Dry matter of shoot and root of tomato plants and (B) Proline content in shoot of tomato plants. G= generation, R= Receptor soil, R+C (receptor mixed with Coppermine Antarctic soil); R+D (receptor mixed with Deception Antarctic soil); R+F (receptor mixed with Fildes Antarctic soil); R+Y (receptor mixed with Yelcho Antarctic soil); R+A (receptor mixed with Arctowsky Antarctic soil). [file DataSheet_1.docx]

**SUPPLEMENTARY TABLE**

| **Soil Sample** | **R** | **Y** | **R+Y** | **C** | **R+C** | **A** | **R+A** | **F** | **R+F** | **D** | **R+D** |
| --- | --- | --- | --- | --- | --- | --- | --- | --- | --- | --- | --- |
| **N (mg kg^-1^)** | 70.0±3.61^a^ | 36.0±2.00^d^ | 58.6±2.52^bc^ | 6.33±1.15^g^ | 29.6±2.08^e*^ | 16.3±1.53^f^ | 62.0±2.00^b^ | 5.67±1.15^g^ | 53.0±2.00^c*^ | 9.67±0.58^g^ | 59.3±1.15^b^ |
| **P (mg kg^-1^)** | 2±0.08^f^ | 179±6.56^a*^ | 37±0.21^c^ | 17±0.58^d^ | 2±0.09^f^ | 43±0.58^b^ | 2±0.08^f^ | 4 ±0.22^f^ | 2±0.08^f^ | 10±0.00^e^ | 2±0.09^f^ |
| **K (mg kg^-1^)** | 131±2.26^c^ | 108±2.26^de^ | 122±2.26^c^ | 200±4.51^b^ | 123±2.26^c^ | 260±4.51^a^ | 74.3±2.26^f*^ | 109±6.77^d^ | 99.1±2.26^e*^ | 100±2.26^de^ | 122±2.26^c^ |
| **pH (H_2_O)** | 5.31±0.00^g^ | 4.36±0.02^h^ | 5.31±0.02^g^ | 7.31±0.03^b^ | 5.51±0.02^e*^ | 6.12±0.03^d^ | 5.43±0.01^f*^ | 8.29±0.02^a^ | 5.39±0.01^f*^ | 7.15±0.04^c^ | 5.42±0.01^f*^ |
| **Organic matter (%)** | 13.2±0.21^d^ | 22.3±0.58^a^ | 16.8±0.31^b*^ | 1.70±0.10^e^ | 12.8±0.25^d^ | 1.20±0.06^e^ | 12.9±0.25^d^ | 1.08±0.03^e^ | 12.6±0.25^d^ | 1.12±0.03^e^ | 14.6±0.67^c^ |
| **K (cmol^+^ kg^-1^)** | 0.34±0.01^c^ | 0.28±0.01^de^ | 0.31±0.01^c^ | 0.51±0.01^b^ | 0.32±0.01^c^ | 0.67±0.01^a^ | 0.19±0.01^f*^ | 0.28±0.02^d^ | 0.25±0.01^e*^ | 0.26±0.01^de^ | 0.31±0.01^c^ |
| **Na (cmol^+^ kg^-1^)** | 0.17±0.01^e^ | 0.45±0.01^cd^ | 0.23±0.01^e*^ | 1.15± 0.07^b^ | 0.42±0.01^cd*^ | 1.65±0.03^a^ | 0.48±0.00^c*^ | 0.42±0.01^cd^ | 0.23±0.00^e*^ | 0.38±0.02^d^ | 0.23±0.00^e*^ |
| **Ca (cmol^+^ kg^-1^)** | 3.04±0.02^f^ | 1.67±0.10^g^ | 3.44±0.02^e*^ | 10.3±0.09^c^ | 3.83±0.03^d*^ | 11.5±0.08^b^ | 3.27±0.01^e*^ | 12.5±0.15^a^ | 3.97±0.01^d*^ | 1.25±0.04^h^ | 3.33±0.01^e*^ |
| **Mg (cmol^+^ kg^-1^)** | 0.97±0.00^h^ | 1.07±0.02f^g^ | 1.02±0.01^gh*^ | 14.6±0.09^a^ | 3.11±0.05^c*^ | 5.90±0.02^b^ | 1.45±0.01^e*^ | 1.76±0.02^d^ | 1.13±0.01^f*^ | 1.05±0.01^fgh^ | 1.04±0.01^fgh*^ |
| **Al (cmol^+^ kg^-1^)** | 0.05±0.00^cd^ | 2.46±0.06^a^ | 0.09±0.00^c*^ | 0.02±0.00^d^ | 0.02±0.01^d*^ | 0.76±0.01^b^ | 0.03±0.00^d*^ | 0.01±0.00^d^ | 0.04±0.00^d*^ | 0.02±0.00^d^ | 0.03±0.00^d*^ |
| **Al Saturation (%)** | 1.10±0.00^d^ | 41.5±0.72^a^ | 1.77±0.01^c*^ | 0.08±0.00^ef^ | 0.30±0.07^ef*^ | 3.70±0.04^b^ | 0.55±0.00^def*^ | 0.07±0.00^f^ | 0.71±0.00^de*^ | 0.68±0.01^def^ | 0.61±0.00^def*^ |
| **ECEC (cmol^+^ kg^-1^)** | 4.56±0.01^h^ | 5.93±0.14^e^ | 5.09±0.02^g*^ | 26.6±0.20^a^ | 7.70±0.03^d*^ | 20.4±0.11^b^ | 5.43±0.01^f*^ | 14.9±0.12^c^ | 5.62±0.01^f*^ | 2.96±0.05^i^ | 4.94±0.01^g*^ |
| **SB (cmol^+^ kg^-1^)** | 4.51±0.01^g^ | 3.47±0.11^h^ | 5.00±0.02^f*^ | 26.56±0.20^a^ | 7.68±0.03^d*^ | 19.7±0.11^b^ | 5.40±0.01^e*^ | 14.9±0.12^c^ | 5.58±0.01^e*^ | 2.94±0.05^i^ | 4.91±0.01^f*^ |
| **WHC (%)** | 66.1±2.40^d^ | ND | 77.2±0.20^b*^ | ND | 73.2±0.80^c^ | ND | 76.5±1.60^bc*^ | ND | 81.4±0.20^a*^ | ND | 82.1±0.40^a*^ |

**Supplementary table 1.** Chemical properties of Andisol soil (Receptor soil; R), Antarctic donor soils (Y, C, A, F and D) and soils mixtures (R+Y, R+C, R+A, R+F and R+D) before multigenerational selection experiment (G0). Values having a common letter are not significantly different (P ≤0.05) according to One-way ANOVA (Tukey test; n=3). ˚ = Plant–available (Olsen) soil P. ND, not determined. * = The means between Andisol soil and mixed soil are statistically different at the P ≤0.05 probability level according to the student’s t test.

**Supplementary table 2**. Statistical determination of community differences (beta diversity) between treatments were evaluated using permutational multivariate analysis of variance (PERMANOVA).

|  |  | Df | Sums_Of_Seqs | MeanSqs | F.Model | R2 | Pr(>F) |
| --- | --- | --- | --- | --- | --- | --- | --- |
| Generation 2 | *Soil* | 5 | 59458 | 11891.5 | 1.9012 | 0.21764 | 0.001 *** |
|  | *Treatment* | 1 | 17218 | 17217.8 | 2.7528 | 0.06303 | 0.001 *** |
|  | *Soil:Treatment* | 5 | 46400 | 9280.1 | 1.4837 | 0.16985 | 0.001 *** |
|  | *Residual* | 24 | 150112 | 6254.7 |  | 0.54948 |  |
|  | *Total* | 35 | 273188 |  |  | 1.00000 |  |
| Generation 5 | *Soil* | 5 | 45574 | 9114.8 | 1.6243 | 0.19428 | 0.001 *** |
|  | *Treatment* | 1 | 16008 | 16008.4 | 2.8528 | 0.06824 | 0.001 *** |
|  | *Soil:Treatment* | 5 | 38324 | 7664.8 | 1.3659 | 0.16337 | 0.001 *** |
|  | *Residual* | 24 | 134673 | 5611.4 |  | 0.57410 |  |
|  | *Total* | 35 | 234580 |  |  | 1.00000 |  |
| Generation 10 | *Soil* | 5 | 60800 | 12160 | 2.0165 | 0.20450 | 0.001 *** |
|  | *Treatment* | 1 | 34459 | 34459 | 5.7143 | 0.11590 | 0.001 *** |
|  | *Soil:Treatment* | 5 | 57323 | 11465 | 1.9011 | 0.19280 | 0.001 *** |
|  | *Residual* | 24 | 144728 | 6030 |  | 0.48679 |  |
|  | *Total* | 35 | 297309 |  |  | 1.00000 |  |

**SUPPLEMENTARY FIGURES**


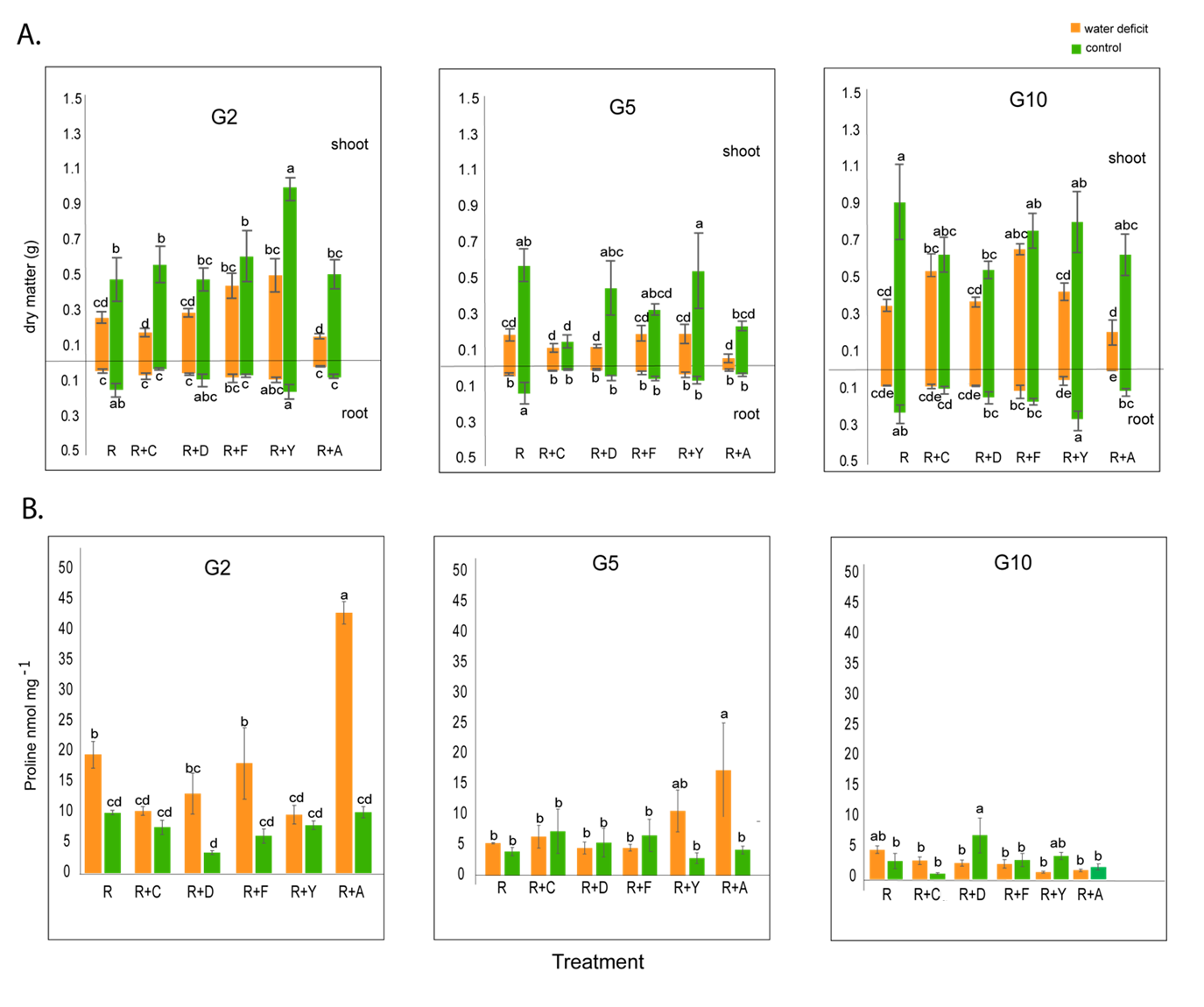


**Figure Supplementary 1**. A. Dry matter of shoot and root of tomato plants and B. Proline content in shoot of tomato plants. G= generation, R= Receptor soil, R+C (receptor mixed with Coppermine Antarctic soil); R+D (receptor mixed with Deception Antarctic soil); R+F (receptor mixed with Fildes Antarctic soil); R+Y (receptor mixed with Yelcho Antarctic soil); R+A (receptor mixed with Arctowsky Antarctic soil).

**
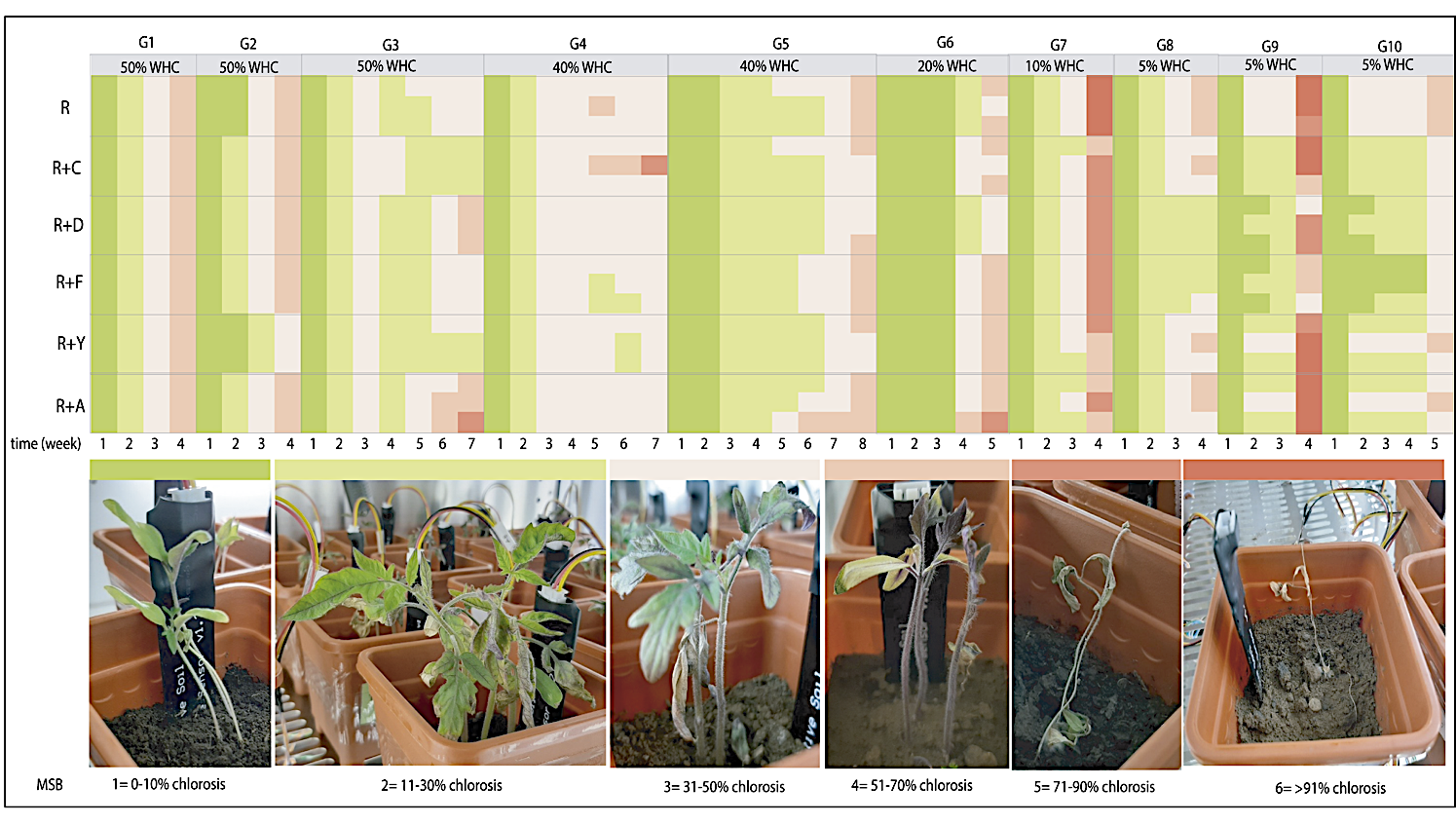
**

**Supplementary Figure 2**. Matrix based scale (MSB) of tomato plants exposed to water deficit stress based to Water-Holding Capacity (WHC) and visualized according to chlorosis scale. From G1 to G3 the water deficit treatments were irrigated at 50% WHC. from G4 to G5 the irrigation decreased to 40% WHC. The G6 was irrigated at 20% WHC and G7 was irrigated at 10% WHC. Finally, from G8 to G10 the plants were irrigated at 5% WHC. G=Generation.

**
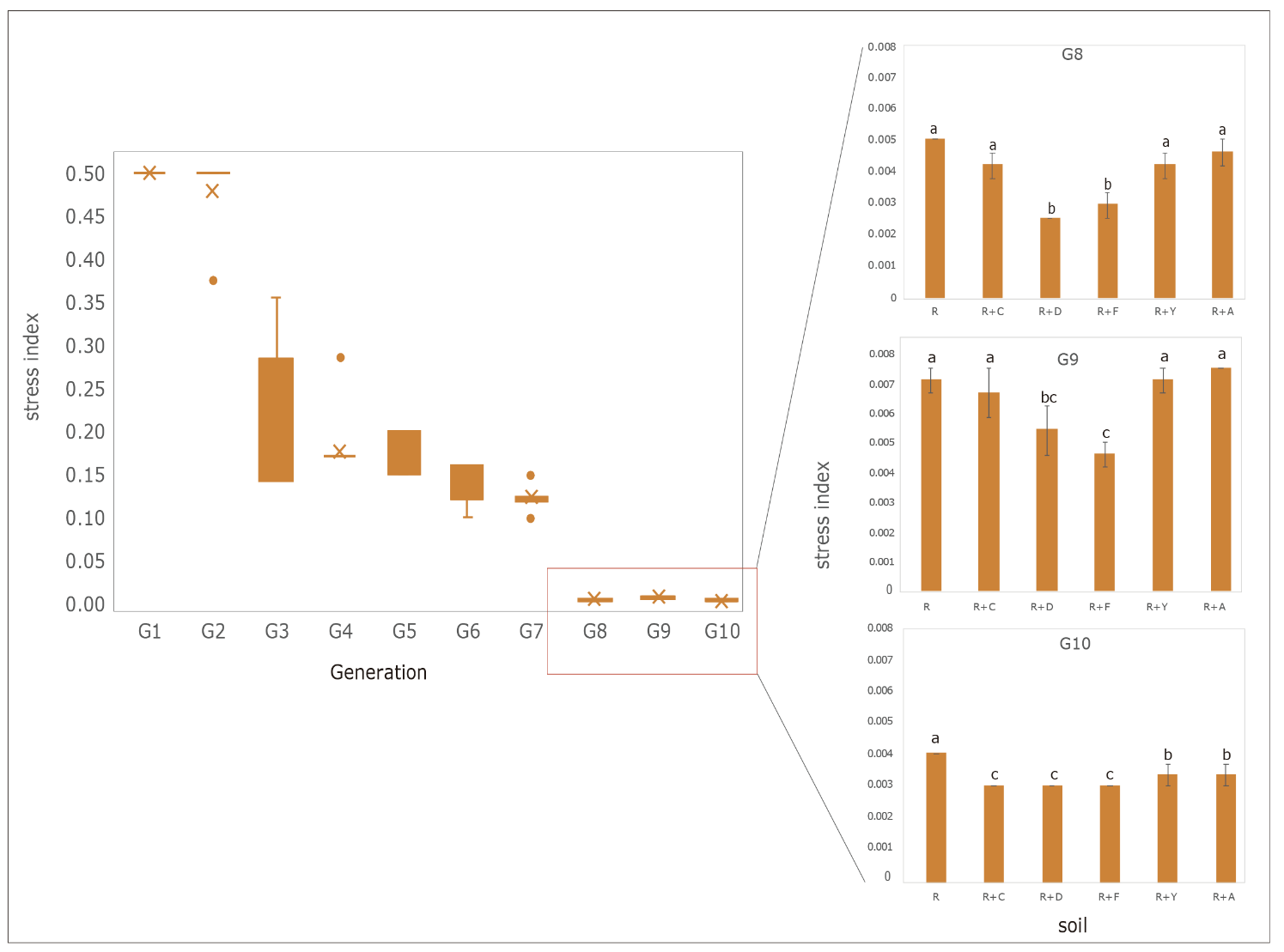
**

**Supplementary Figure 3**. Stress index obtained from the MSB taking account the supplementary equation 1.

**
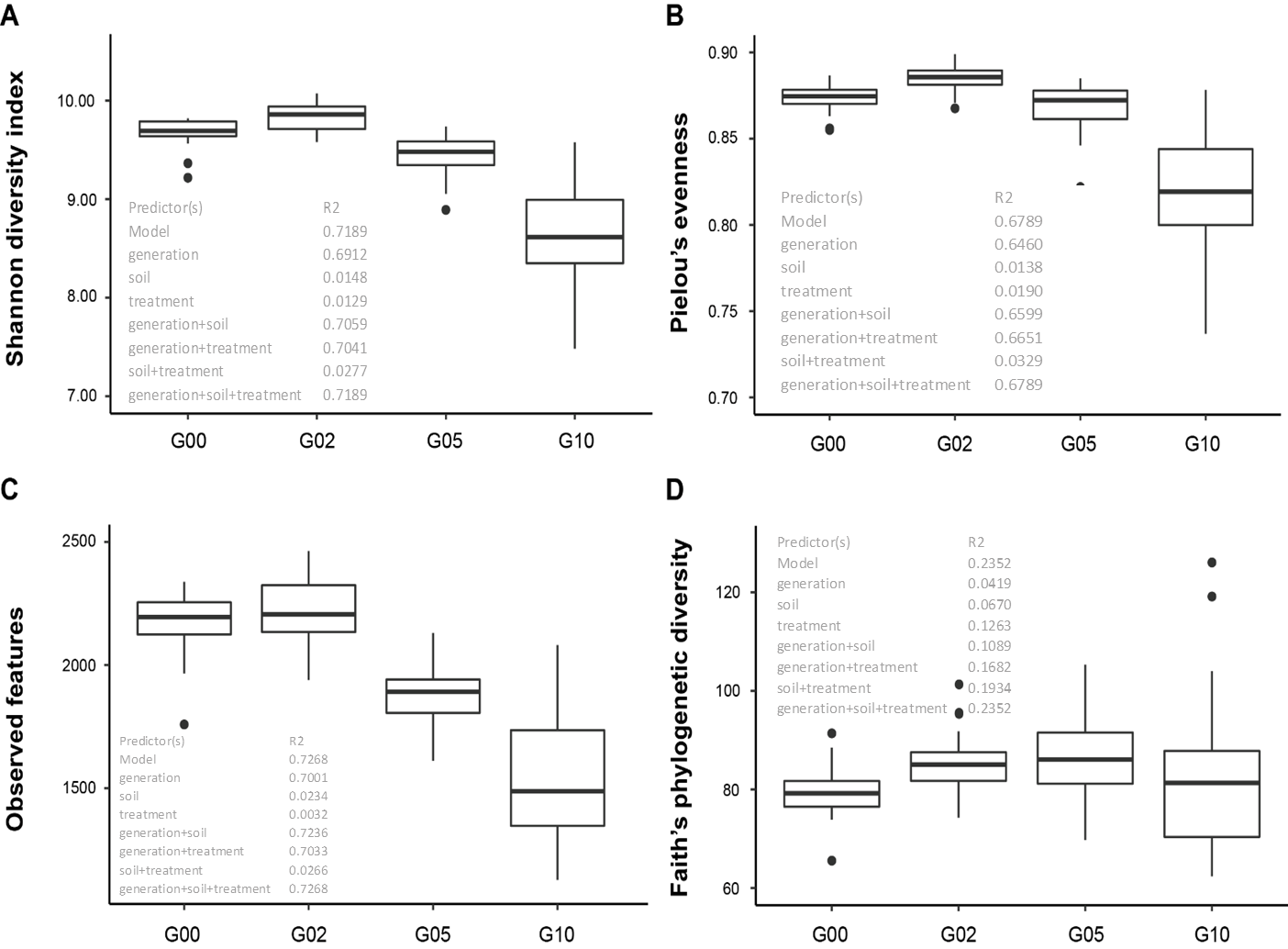
**

**Supplementary Figure 4.** Alpha diversity of all soil samples**.** (A) Shannon's diversity index, (B) Pielou´s evenness, (C) Features (richness) and (D) Faith's Phylogenetic Diversity of all data and all generations.


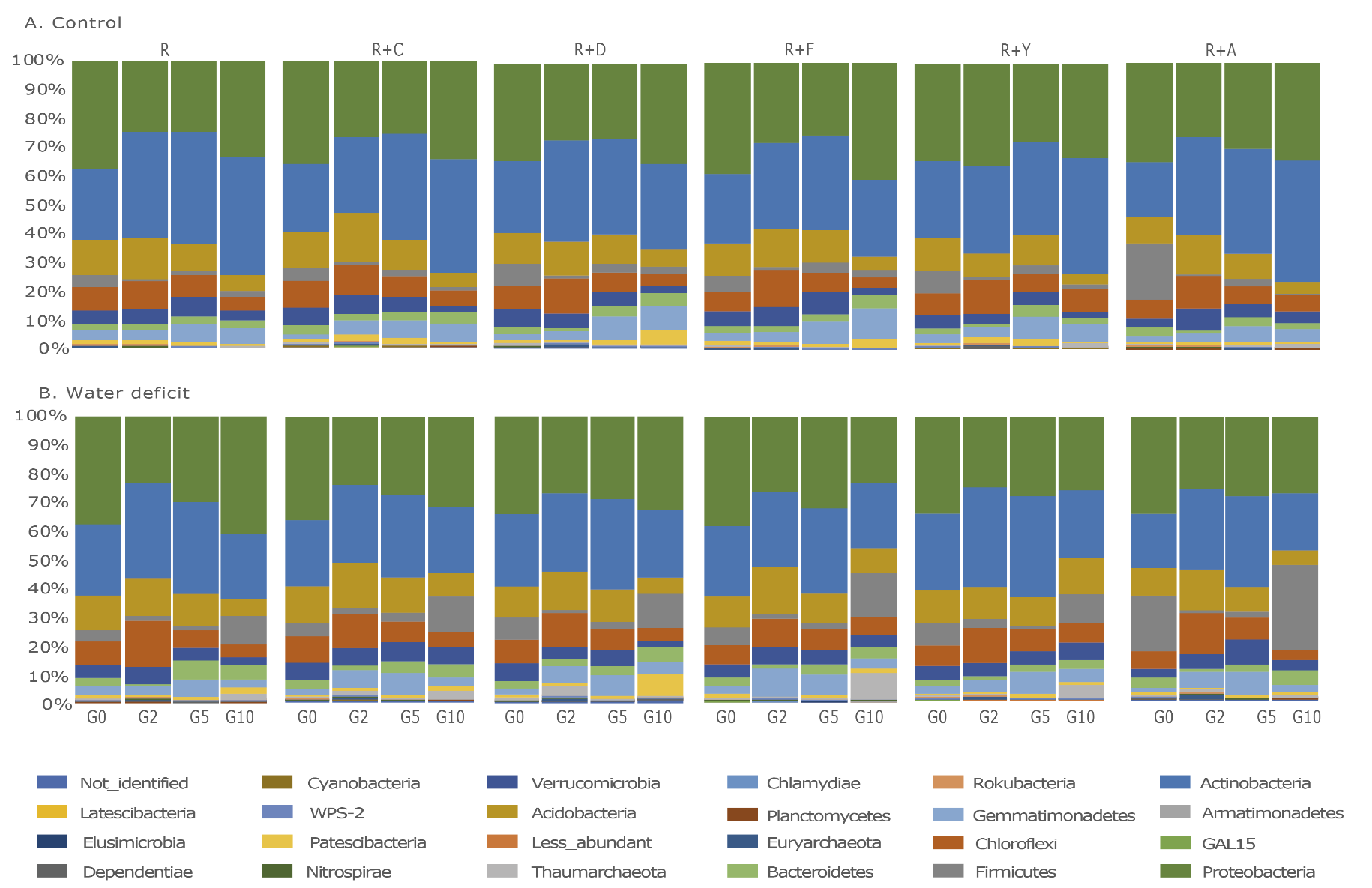


**Supplementary figure 5.** Relative abundance of each taxa at the phylum level in the total rhizobacterial community associated with G2, G5 and G10 in water deficit and control treatments. G=generation.


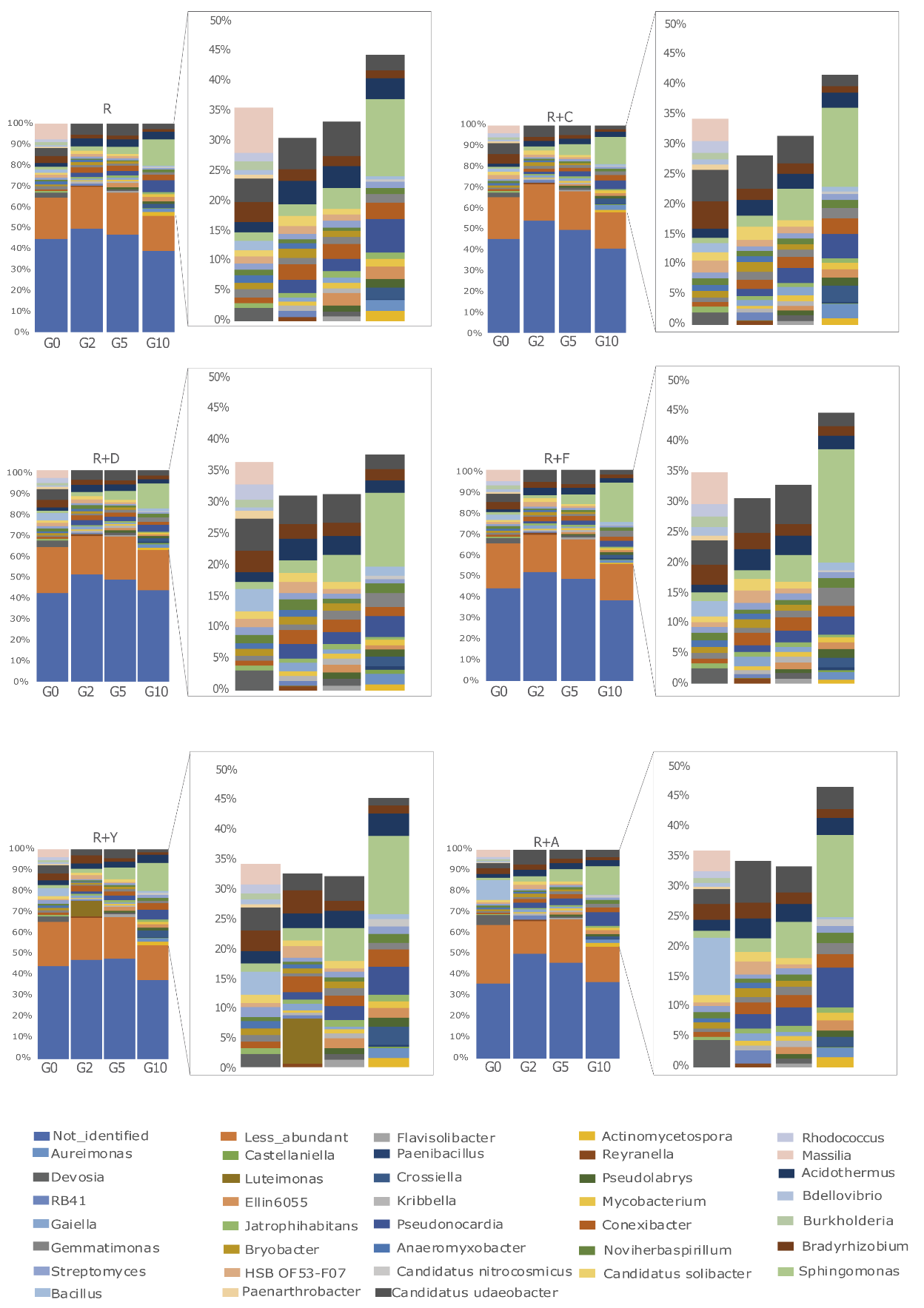


**Supplementary Figure 6**. Relative abundance of each taxa at the genus level in the total rhizobacterial community associated with G2, G5 and G10 in control treatments. G=generation.


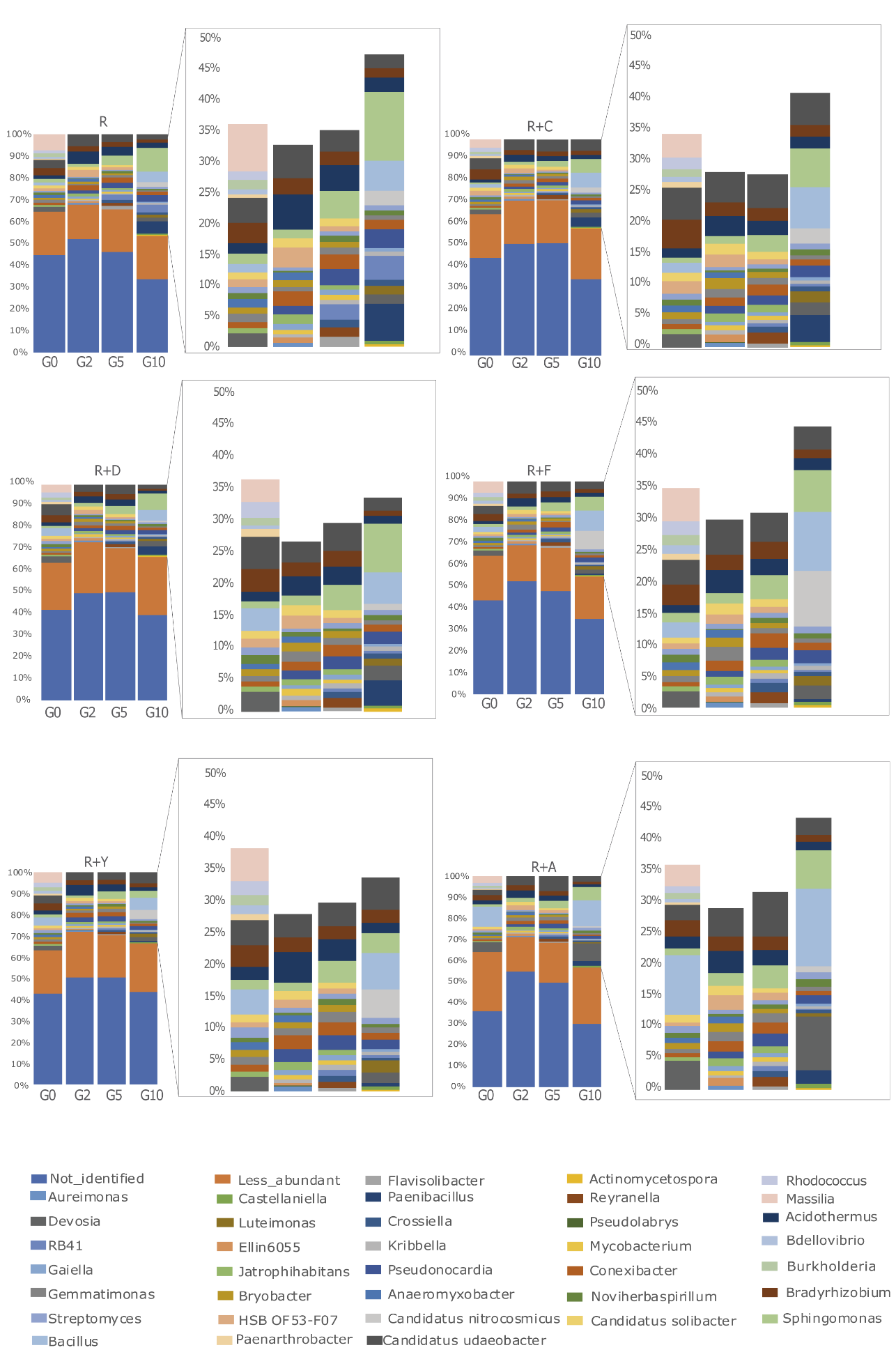


**Supplementary Figure 7.** Relative abundance of each taxa at the genus level in the total rhizobacterial community associated with G2, G5 and G10 in water deficit treatments. G= generation


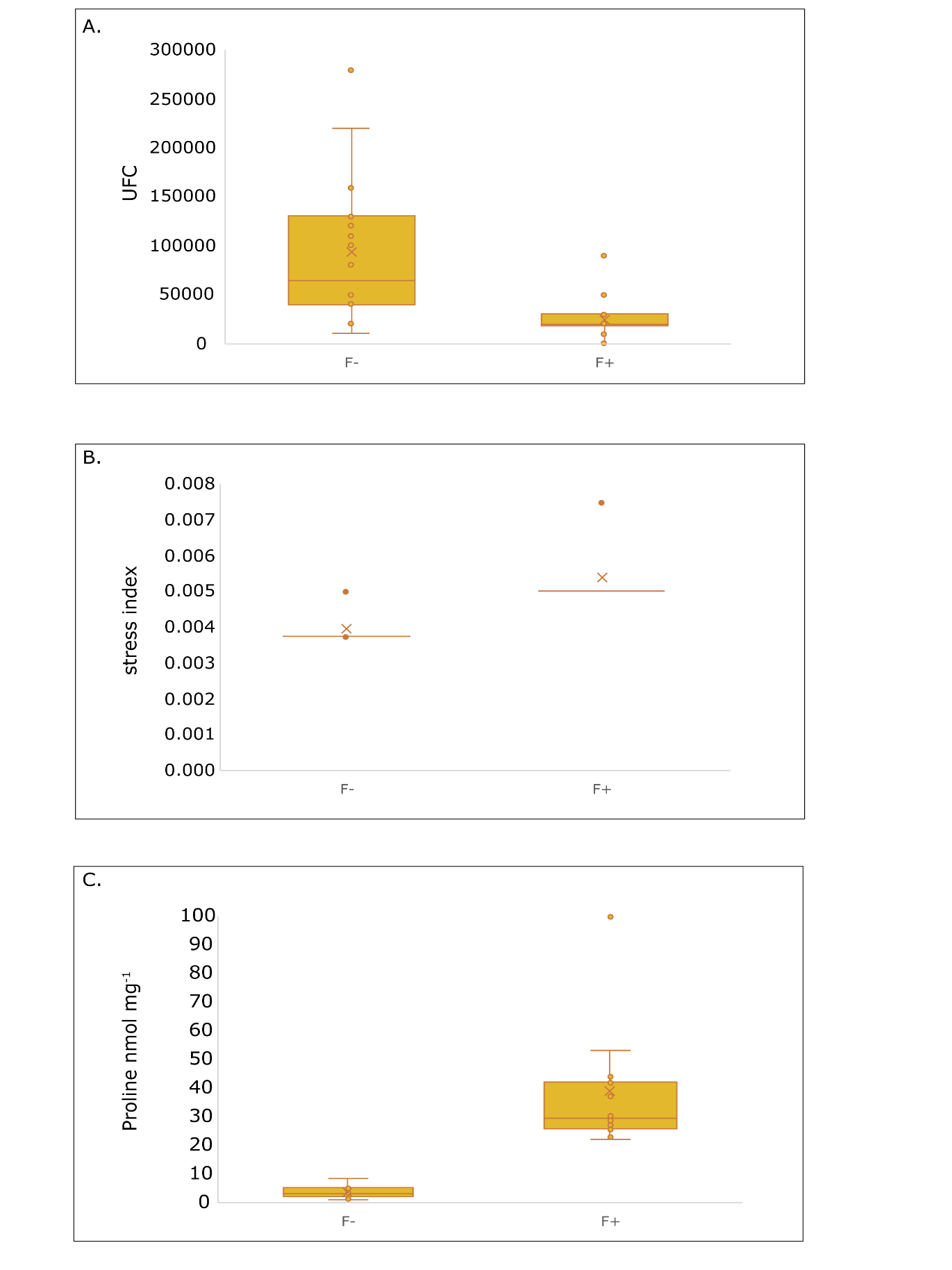


**Supplementary Figure 8.** Unit formed of colony (A), stress index obtained from the MSB taking account the equation 1, G= generation (B) and Proline content on fumigated (F+) and not fumigated (F-) treatments with chloroform.


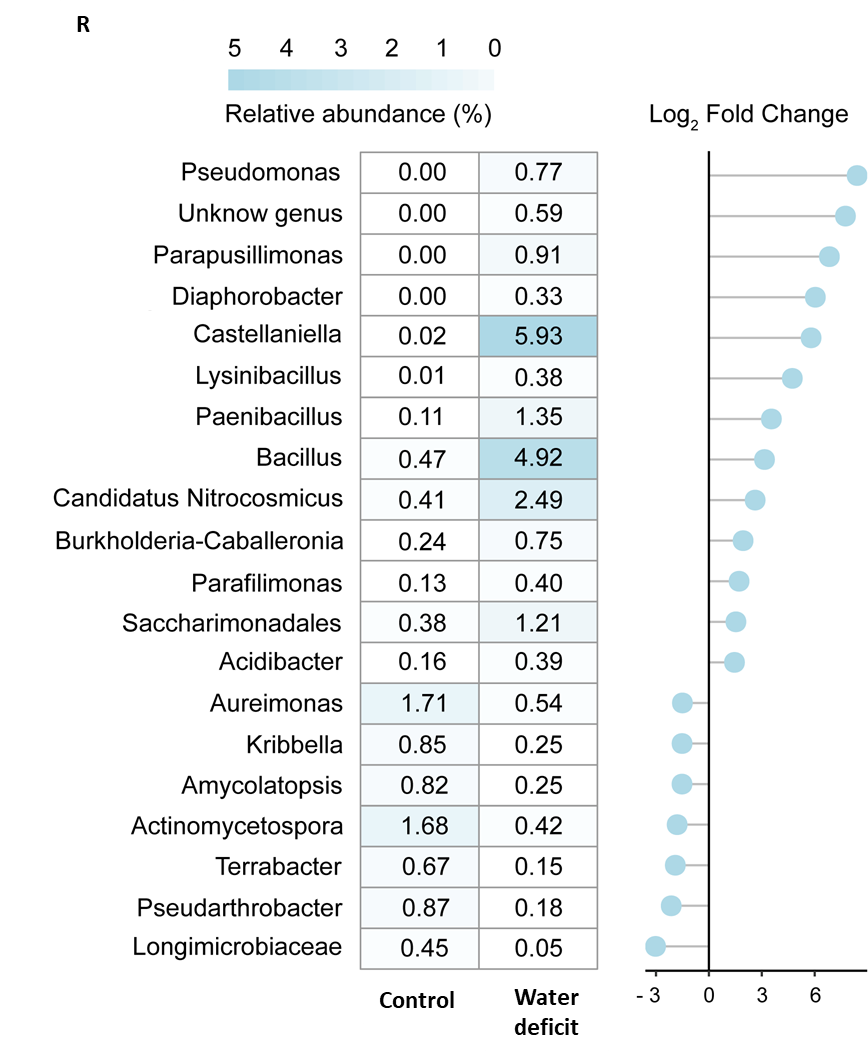


**Supplementary Figure 9**. Differential abundance by ZIGMM using metabarcoding data in G10 of R treatment. Negative values in Log_2_ Fold change represent a loss in the relative abundance of the species and positive values in Log_2_ Fold Change represent gains in relative abundance for each taxonomic group.


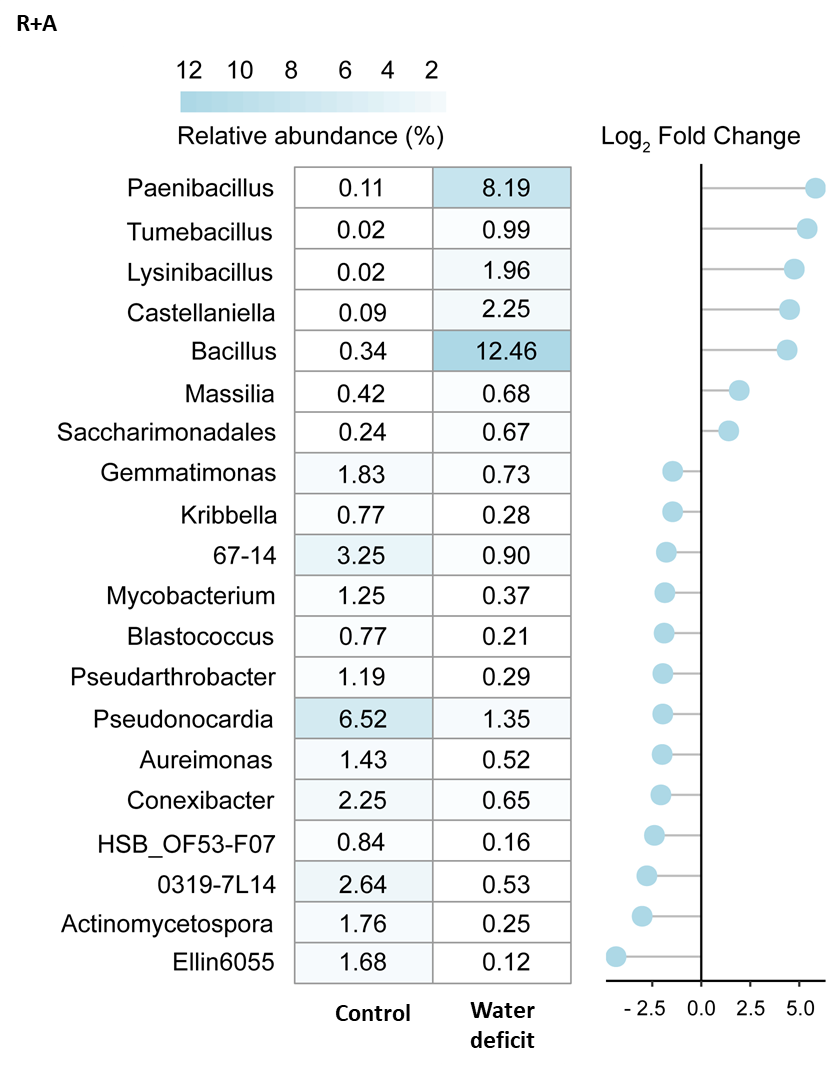


**Supplementary Figure 10**. Differential abundance by ZIGMM using metabarcoding data in G10 of R+A treatment. Negative values in Log_2_ Fold change represent a loss in the relative abundance of the species and positive values in Log_2_ Fold Change represent gains in relative abundance for each taxonomic group.


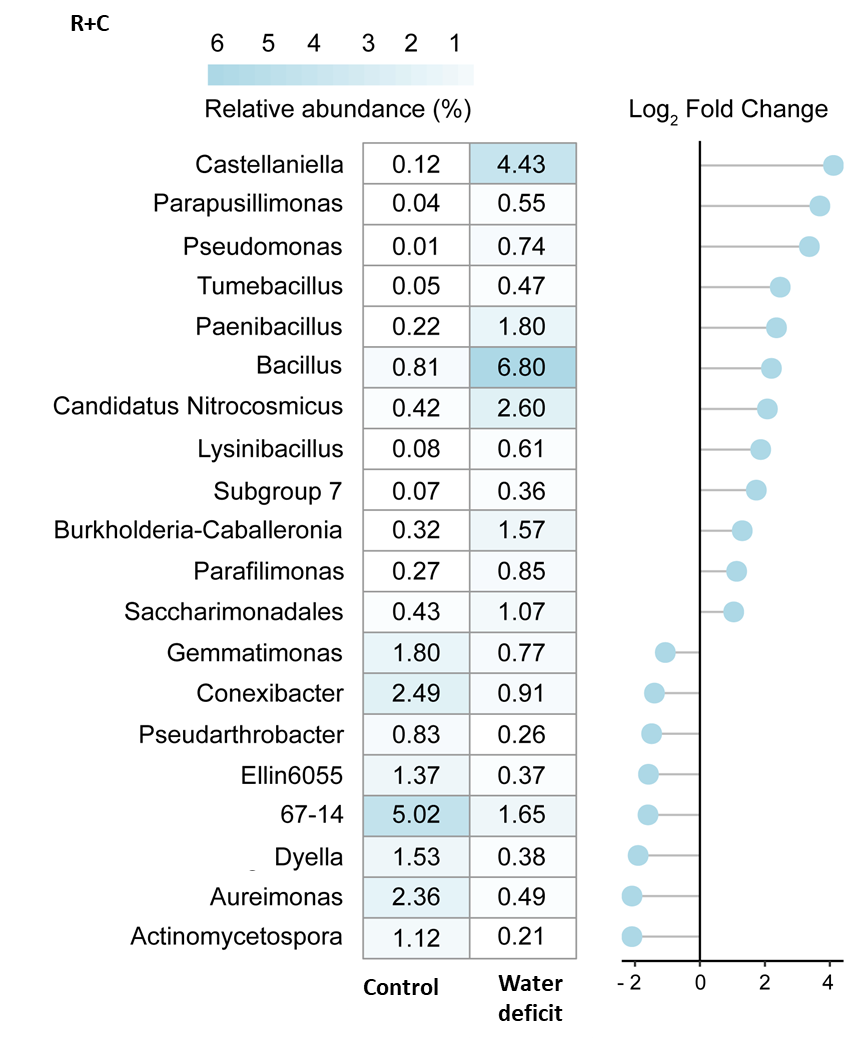


**Supplementary Figure 11**. Differential abundance by ZIGMM using metabarcoding data in G10 of R+C treatment. Negative values in Log_2_ Fold change represent a loss in the relative abundance of the species and positive values in Log_2_ Fold Change represent gains in relative abundance for each taxonomic group.

**
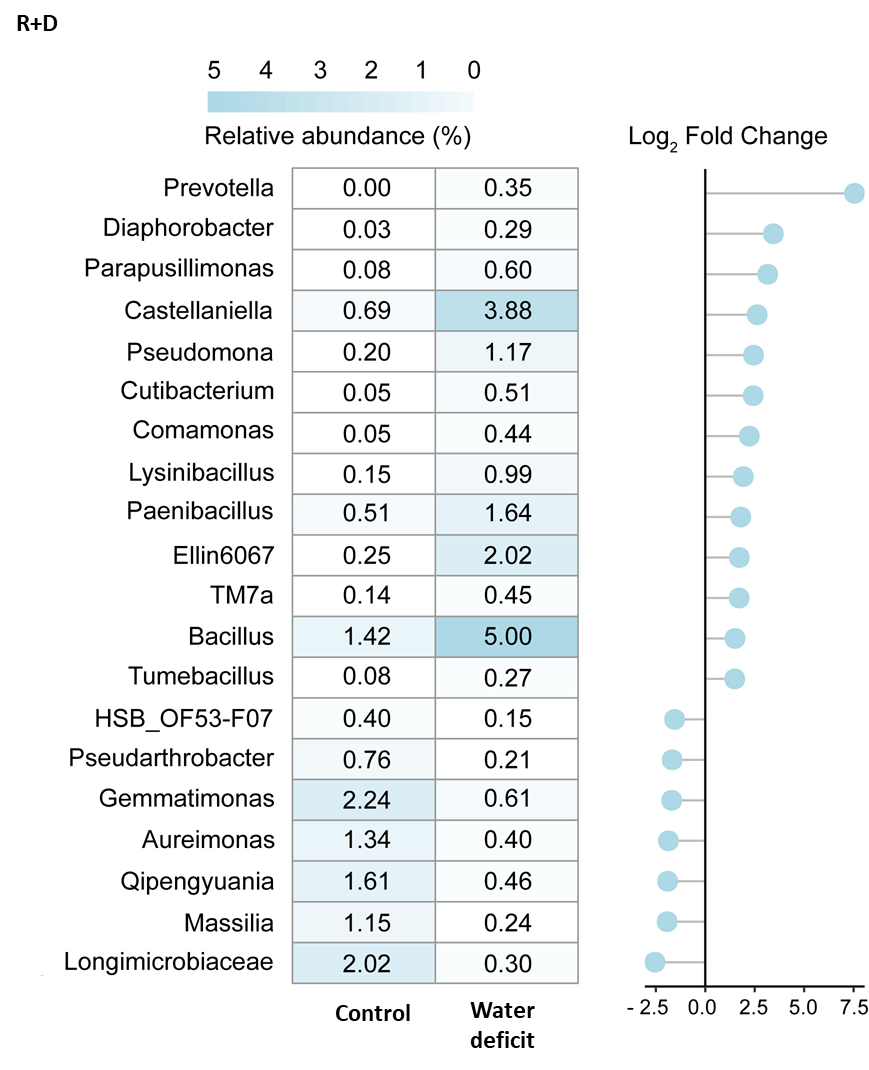
Supplementary Figure 12**. Differential abundance by ZIGMM using metabarcoding data in G10 of R+D treatment. Negative values in Log_2_ Fold change represent a loss in the relative abundance of the species and positive values in Log_2_ Fold Change represent gains in relative abundance for each taxonomic group.


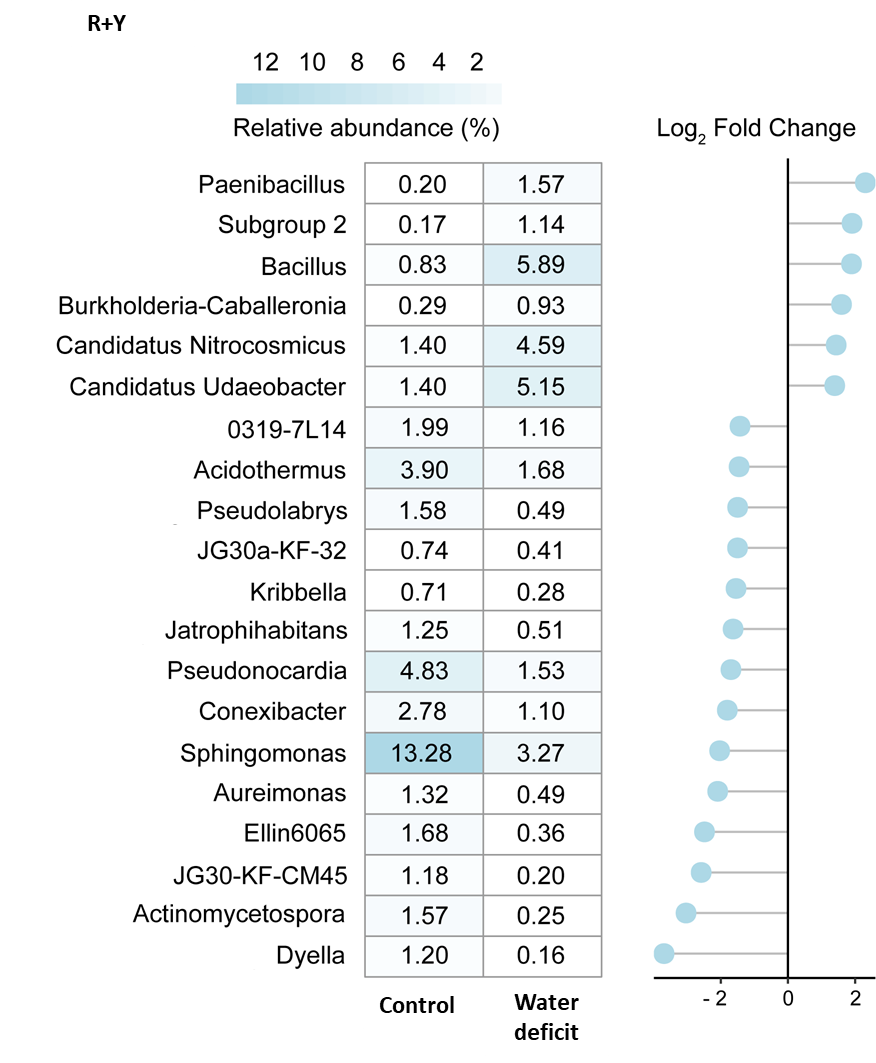


**Supplementary Figure 13**. Differential abundance by ZIGMM using metabarcoding data in G10 of R+Y treatment. Negative values in Log_2_ Fold change represent a loss in the relative abundance of the species and positive values in Log_2_ Fold Change represent gains in relative abundance for each taxonomic group.

**SUPPLEMENTATY EQUATION**

$Stress index=\sum_{n=3}^{n} \left( \frac{MSB}{Time(weeks)} \right)\times\% WHC$ *Supplementary Equation 1*

Where, MSB is a Matrix Scale-Based assignment, %WHC is the water holding capacity in the generation studied and the Time (weeks) is the corresponds to the survival time of the plants in that generation.
